# Design of an artificial phage-display library based on a new scaffold improved for average stability of the randomized proteins

**DOI:** 10.1038/s41598-023-27710-4

**Published:** 2023-01-24

**Authors:** M. Gomes, A. Fleck, A. Degaugue, F. Gourmelon, C. Léger, M. Aumont-Nicaise, A. Mesneau, H. Jean-Jacques, G. Hassaine, A. Urvoas, P. Minard, M. Valerio-Lepiniec

**Affiliations:** 1 Université Paris-Saclay, CEA, CNRS, Institute for Integrative Biology of the Cell (I2BC), 91198 Gif-sur-Yvette, France; 2 Arcoscreen, École Polytechnique Fédérale de Lausanne (EPFL), Lausanne, Switzerland

**Keywords:** Proteins, Molecular engineering

## Abstract

Scaffold-based protein libraries are designed to be both diverse and rich in functional/folded proteins. However, introducing an extended diversity while preserving stability of the initial scaffold remains a challenge. Here we developed an original approach to select the ensemble of folded proteins from an initial library. The thermostable CheY protein from *Thermotoga maritima* was chosen as scaffold. Four loops of CheY were diversified to create a new binding surface. The subset of the library giving rise to folded proteins was first selected using a natural protein partner of the template scaffold. Then, a gene shuffling approach based on a single restriction enzyme was used to recombine DNA sequences encoding these filtrated variants. Taken together, the filtration strategy and the shuffling of the filtrated sequences were shown to enrich the library in folded and stable sequences while maintaining a large diversity in the final library (Lib-Cheytins 2.1). Binders of the *Oplophorus* luciferase Kaz domain were then selected by phage display from the final library, showing affinities in the μM range. One of the best variants induced a loss of 92% of luminescent activity, suggesting that this Cheytin preferentially binds to the Kaz active site.

## Introduction

Antibodies have consistently dominated the field of specific protein binding reagents in both fundamental research and therapeutic applications^1,2^. In the past two decades, new molecular recognition repertoires based on small globular proteins were created to bypass known limits of antibody fold. Highly diverse libraries of protein variants have been assembled and the rare variants of the library able to bind pre-defined targets can be selected by using powerful protein interactions screening methods, such as phage display or related methods. These new libraries are either based on nanobodies (VHH)^3^ or on scaffold proteins not related to antibodies^4–6^. Synthetic protein libraries present a number of advantages. First, no animal immunization is required as large naïve libraries are efficient sources of binders^3^. Second, selected binders can be efficiently produced in prokaryotic expression systems and easily engineered by fusion with other proteins. Third, cysteine and disulfide bond free scaffolds allow to introduce unique cysteines in appropriate positions for targeted conjugation to small molecules such as fluorescent dyes. Finally, such specific binders offer innovative possibilities such as new protein purification tools and crystallization helpers^7,8^, tools for target-tracking or degradation in living cells^9,10^, or biosensors and medical-imaging tools^11^ or drugs candidates^4–6,12–14^.

Although the general utility of alternative scaffold libraries is now well established, the design and assembly of an efficient library is still challenging. Consequently, only a few protein libraries have so far been demonstrated to be a generic source of specific binders for almost any protein target of interest^6^. In fact, the most fundamental difficulty in designing an efficient protein library is to find a compromise between two opposite requirements: on one hand, the number of randomized amino acids must be sufficient to create a potential binding surface, but on the other hand, multiple random changes in the amino acids are more likely to drastically affect protein stability^15–18^.

Biological input might help to satisfy these contradictory requirements and to design efficient libraries. For example, using a thermostable domain as starting scaffold helps to preserve a sufficient stability in variegated sequences. Furthermore, protein families that have naturally evolved for versatile binding capacities can help to design a library with an efficient randomization scheme. Proteins made from structural repeats are in this respect particularly attractive as these families have naturally evolved from simple motifs to give rise to versatile binding architectures. Sequence analysis of a natural repeat family allows to clearly delineate the repertoire of accepted side chains for each position of the repeated motif. Based on these principles, highly efficient protein libraries based on idealized repeats were designed; DARPins^19^ based on ankyrin motifs were first described and followed by other highly efficient libraries such as alphaRep^20^ based on HEAT repeats or Repebodies based on Leucine rich repeats^14^.

In the more general case of non-repeated sequences, the design of libraries is often empirical both for the choice of the positions to be randomized^21^ and for the set of side chains allowed at each randomized position^22^.

The fundamental questions underlying library design were more recently systematically addressed by combining high throughput selections using yeast display and large-scale sequence analysis of selected sequences pools. It was then possible to evaluate systematically the scaffold properties and diversification schemes resulting in optimized libraries^23,24^. Similar high throughput methods were used to identify high affinity binders from designed scaffolds^25^ as well as scaffolds with favorable developabilty^23,26^.

Here, we have investigated a new and potentially general strategy to improve the quality of a protein library based on an alternative scaffold. The general idea is to select from the initial library the subset of diversified sequences which are correctly assembled at the DNA level and more importantly that effectively give rise to folded proteins. This “filtrated” subset can then be recombined using an efficient single step shuffling procedure, to create a new diverse library enriched in fold-compatible sequences. We herein show that recombination of pre-selected folded proteins is an efficient way to improve the proportion of folded and stable variants in the diverse library.

One of our objectives was to generate a new synthetic inhibitor of nano-Kaz luciferase, a luminescence-generating enzyme derived from the *Oplophorus* luciferase. In the near future, such an inhibitor is promising to become an essential component of new biosensors based on a ligand-induced conformational switch^27,28^. We therefore used this improved new library to generate a specific binder acting as functional inhibitor of Kaz luciferase.

## Results

### Choice of the template protein and identification of residues for randomization

**Figure 1:**
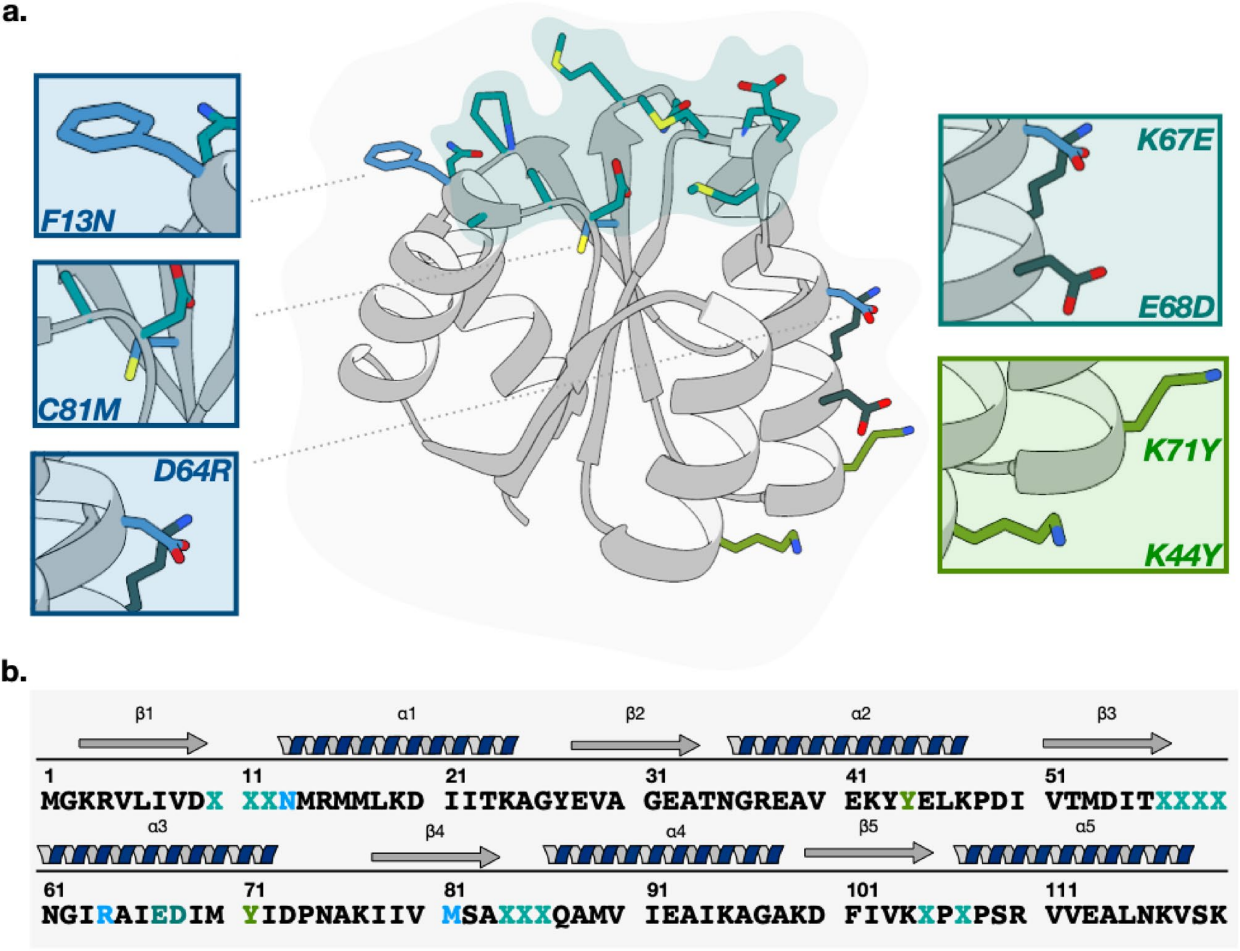
Design of the Cheytins Library. (**a**) The 3D XRay-structure CheY (pdb: 2TMY): a scaffold to design a novel library. The backbone corresponding to the constant residues is represented in grey. The randomized residues are displayed in cyan stick in the four exposed loops. The corresponding surface is also highlighted in cyan. The mutations R13N, D64R, C81M corresponding to the stability of the scaffold are shown in light blue stick. The two mutations K67E and E68D corresponding to the insertion of BbsI restriction sites in the CheytinWT are represented in dark green stick and the mutations K44Y and K71Y are shown in light green. (**b**) Amino acid sequence of the CheytinWT with residues represented with the same colors as in the structure. Randomized positions are represented by a blue cross in position 10, 11, 12, 57, 58, 59, 60, 84, 85, 86, 105, 107.

CheY, a response regulator, was chosen as initial scaffold to construct the library. Response regulators are proteins involved in prokaryotic two-components transduction systems and have evolved as versatile protein interaction modules regulated by phosphorylation^29^. The CheY (pdb: 2TMY) protein from the thermophilic microorganism *Thermotoga maritima*^30,31^ has been characterized in detail. It is a relatively small (125 aa, 13.2 kDa) monomeric and highly thermostable (Tm = 95 °C) protein. The protein is efficiently expressed in *E. coli* (20 mg of Che Y protein/100 mg of protein from the cytoplasmic supernatant). The overall fold, common to all phosphoacceptor receiver domain (REC) of response regulators proteins is made from a five-stranded parallel β-sheet surrounded by five α-helices (Fig. 1a). The exposed loops are involved in mediating the interaction both with the upstream kinase and the downstream effector domain of response regulators^32^. Due to their biological function, these loops are accessible for interaction with protein partners. For randomization, we selected four structurally contiguous beta-to-alpha connecting loops as the flexible shape of this surface appears well-adapted to fit in pockets such as active sites of enzyme^33,34^.

To build the novel library based on CheY, 12 residues were identified to be randomized corresponding to positions 10, 11, 12, 57, 58, 59, 60, 84, 85, 86, 105, 107 in the protein final sequence (Fig. 1b). Some mutations were also introduced in the CheY sequence (GenBank: AAA96389) before randomization: the previously described substitutions R13N and D64R were introduced to stabilize the CheY-fold^35^. Cysteine 81 was replaced by a Methionine to prevent improper disulphide bond formation in the protein variants. Other substitutions were introduced for the need of library construction: substitutions K67E and E68D allowed the introduction of a BbsI restriction site essential for the recombination strategy (described below). Two mutations, K44Y and K71Y, were also introduced on the external surface of the protein, to provide another interaction surface with other synthetic protein partners, although this property is not used in the present work.

We named these CheY-derived proteins, Cheytins. The first resulting protein, including all these substitutions, called CheytinWT is very stable with a Tm, obtained by DSC, of 77.90 ± 0.01 °C (see supplementary Fig. S1). CheytinWT is thus well suited to be used as a scaffold for Cheytin variants library construction (Fig. 1).

### Randomization characteristics

Randomization was implemented using synthetic degenerated oligonucleotides based on trinucleotide cassettes^36,37^. Unlike other methods, such as commonly used NNK or NNS degenerate codons, this approach minimizes the codon bias, eliminate all stop-codons and allows to incorporate at each position a predefined subset of amino acids. Here a mixture of 19 trinucleotide phosphoramidites was prepared encoding all amino acids except cysteine.

The frequencies distribution of variable sidechains was biased to enhance the average interaction propensity of the randomized surface and was based on the sidechain distribution observed in the human and murine antibody loops CDR-H3 in which tyrosine, serine and glycine residues are overrepresented^38^. Indeed, the third CDR loop of the heavy chain (CDR-H3) has been shown to play an essential role in antibody-antigen interactions, since it often constitutes the determinant of antibody specificity and affinity^39–41^. Furthermore, similar strategies have already shown successful results in other artificial protein libraries^4,42^. The mixtures of trinucleotides (supplementary Table S2) were thus settled according to the chosen frequencies and applied in identical conditions to all randomized positions. The sampled diversity was enriched specifically in tyrosine (frequency = 25%), glycine (18.5%), serine (8.5%), alanine (6.5%), aspartate (6.5%). The frequency adopted for the other residues is maintained at 2.5%^38^.

### Construction of an optimized Library

#### Construction of the initial library Lib-Cheytins 1.0

**Figure 2:**
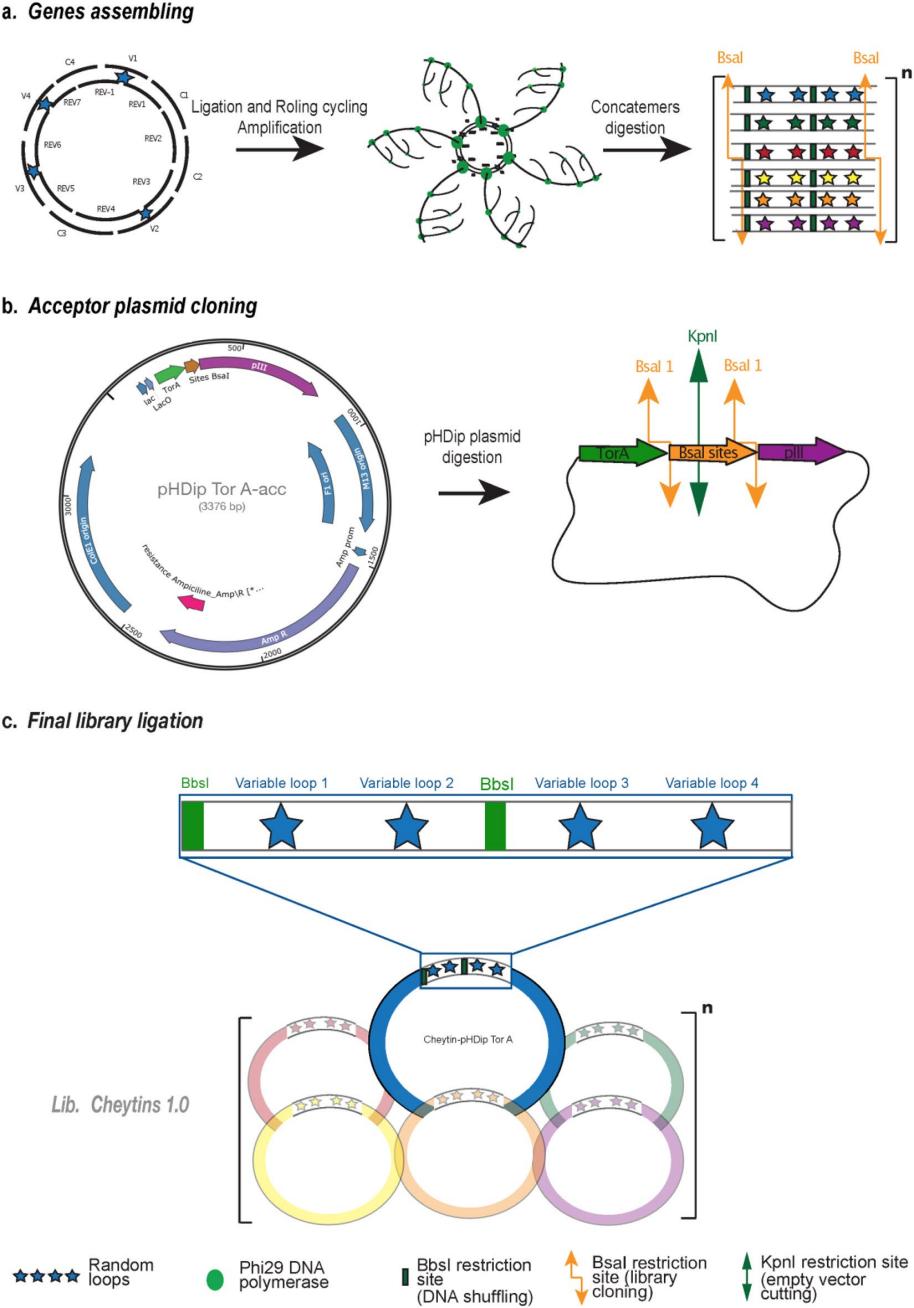
Cheytins-library construction. (**a**) The sequence corresponding to the CheytinWT protein was coded by a set of oligonucleotides including four variable degenerate oligonucleotides (V1–V4); four constant, non-degenerate oligonucleotides (C1–C4); degeneration is indicated by stars and corresponds to amino acid positions 10, 11, 12, 57, 58, 59, 60, 84, 85, 86, 105, and 107 in the sequence of the CheytinWT. (Detailed oligonucleotide sequences are given in Materials and Methods). The oligonucleotides were assembled with eight reverse primers (Rev1–Rev7 + Rev-1) and ligated to form DNA circles coding for the Cheytin sequence. The circles were used as a matrix for RCA amplification to generate double-stranded homopolymers of Cheytin variants. Digestion of the RCA products with a restriction enzyme (BsaI) gave rise to a collection of single Cheytin variants with different sequences. (**b**) A detailed view of the acceptor vector pHDip TorA. The acceptor vector (pHDip Tor A -acc) used for the library construction contains a cassette with two BsaI restriction sites. BsaI digestion of this vector generates compatibles ends with the insert encoding Cheytin variants obtained after RCA product digestion with BsaI. Each phagemide of the library is circularized by ligation. (**c**) Final assembly of the library: The sequences of the Cheytin variants were ligated in acceptor vector (pHDip Tor A- acc) previously digested at KpnI and BsaI restriction sites. The KpnI site was used to prevent recircularization of the acceptor vector throughout ligation.

A first library was constructed using a rolling circular amplification-based technology^20,43,44^ (RCA). The oligonucleotides corresponding to variable and constant sequences were assembled and ligated together with the reverse oligonucleotides to produce double-stranded circular sequences. The circular DNA fragments were amplified by RCA, digested with BsaI and sub-cloned into the empty phagemid (Fig. 2a–c).

Preliminary measurements of Cheytin-display efficiency using different signal sequences involved in the translocation through the SEC and TAT pathways were conducted (Fig. S2)^45^. It appeared that the most efficient display for the CheytinWT was observed with the TorA signal sequence involved in the TAT export system (Tween Arginine Translocation). The TorA sequence, was thus chosen as an export sequence in the phage display construction.

A first library, named Lib-Cheytins 1.0, containing 8.1 × 10^7^ independent clones was obtained. The sequence of a preliminary pool of 20 randomly picked clones indicated that 55% of them (corresponding to 4.5 × 10^7^ in the Lib-Cheytins 1.0) display the expected in-frame nucleotide sequences while the remaining 45% encoded incorrect sequences (errors in the oligonucleotide assembly and/or frame shift in the sequences).

#### New enrichment procedure to capture the ensemble of folded variants

**Figure 3:**
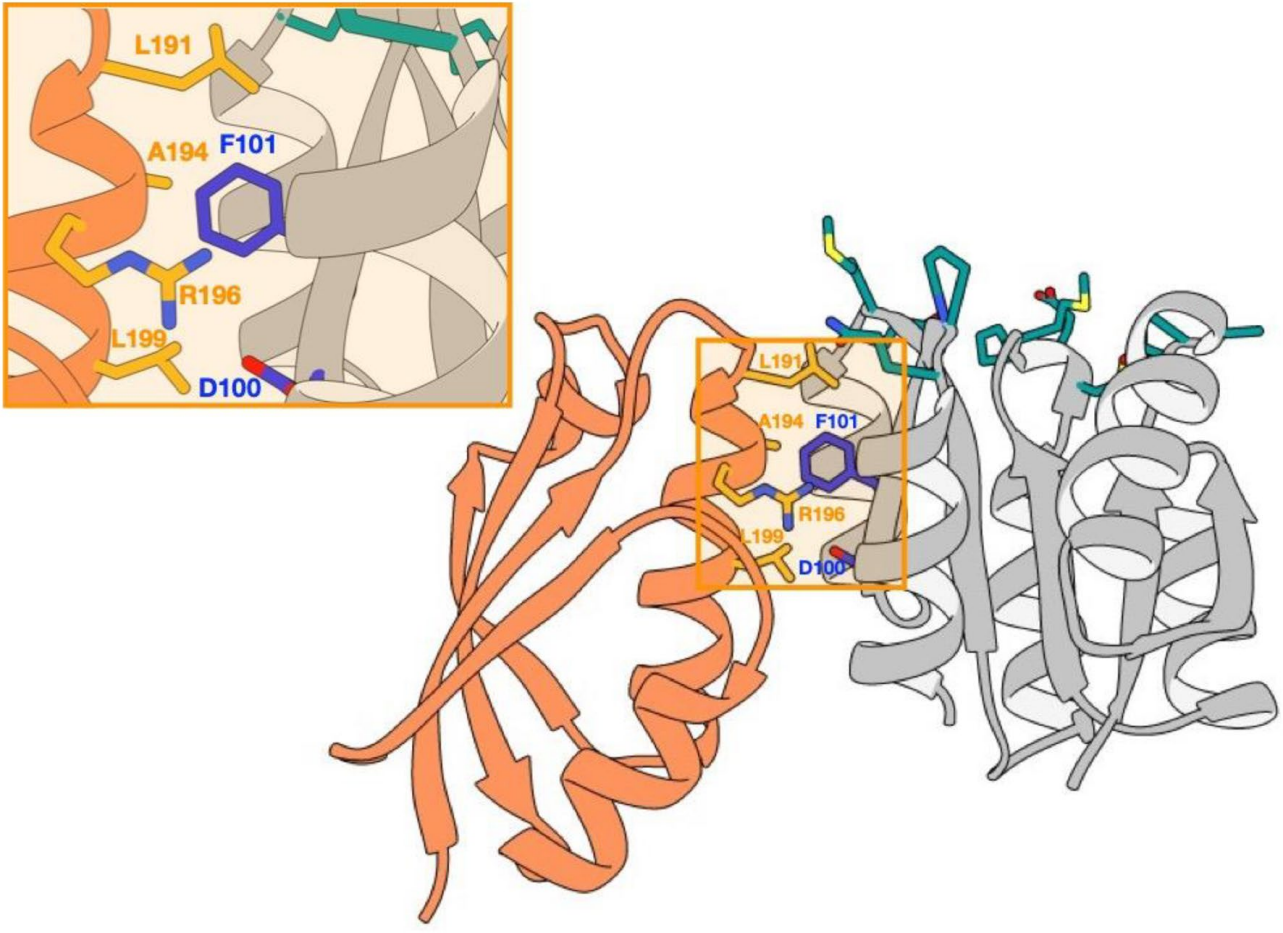
Complex between CheY and its biological partner the P2 domain of CheA. In the crystallographic structure of the CheY/P2 complex of *T. maritima* (PDB code: 1U0S), P2 protein (left) is shown in orange, CheY protein (right) is represented in grey and the randomized residues in cyan stick in the four exposed loops as in Fig. 1a. The residues involved in the interaction between P2 and CheY are highlighted in yellow stick for the P2 domain (L191, A194, R195 and L198) and in dark blue for CheY (D100 and F101)^47^.

The proportion of incorrect sequences remained high in the Lib-Cheytins 1.0 and had to be improved. Furthermore, an unknown proportion of coding nucleotide sequences could result in highly destabilized proteins, unable to give rise to specific binders. To solve both of these potential difficulties an innovative “conformational filtration” procedure based on phage display selection with a specific protein partner was devised. The CheY protein has a biological partner CheA composed of 5 domains P1–P5^46^. The P2 domain (PDB code: 1U0S_A) is directly involved in the interaction with CheY. Its interaction surface with CheY is distinct from the randomized loops surface (Fig. 3)^47^. Our expectation was therefore that only correctly folded proteins from Lib-Cheytins 1.0 displayed on the surface of the M13 bacteriophage should be captured by P2 immobilized on an ELISA plate. Phages exposing truncated proteins, unfolded proteins or that do not display proteins should not be selected.

The CheytinWT was first tested for its ability to bind P2 protein. A K_D_ of 0.79 ± 0.09 µM measured by ITC showed that the mutations introduced in the CheY to generate the library-template protein CheytinWT, did not disrupt the affinity to P2, although it was decreased by a factor of 5.2 (Fig. S3). Then, the filtration, based on the interaction of P2 with correctly folded variants in the library, gave rise to an ensemble of 2.8 × 10^6^ independent clones (Lib-Cheytins 2.0). Sequence analysis of 40 clones randomly picked in this pool of filtrated proteins showed a significant increase in correct in-frame sequences from 55 to 90% (36/40). This ‘‘filtrated’’ library, called Lib-Cheytins 2.0, finally contains 2.5 × 10^6^ folded clones (90% of 2.8 × 10^6^). Proteins produced by four coding variants randomly picked in the LibCheytin 2.0 were purified and tested by ITC for their ability to bind P2. These four Lib-Cheytins 2.0 variants bind P2 with a K_D_ ranging from 0.11 to 0.66 µM (Fig. S3).

#### Recovering diversity in the library by DNA shuffling

About 2.5 × 10^6^ folded variants were thus recovered in the library Lib-Cheytins 2.0. To compensate for the diversity loss resulting from the “conformational filtration” (4.5 × 10^8^ variants in Lib-Cheytins 1.0 down to 2.5 × 10^6^ variants in Lib-Cheytins 2.0) the ensemble of preselected sequences was split in fragments and then recombined using a shuffling procedure. This shuffling process recombined the fragments from a subset of the initial library that comprises only folded sequences. All strongly destabilized sequences are eliminated from this subset. The underlying assumption is that recombination of fold compatible sequences should give rise to a highly diverse library with an improved fraction of foldable sequences. The theoretical diversity re-created by recombination of two fragments from a library of 10^6^ independent clones is 10^12^ which is much higher than the experimental diversity of phage display libraries.

**Figure 4:**
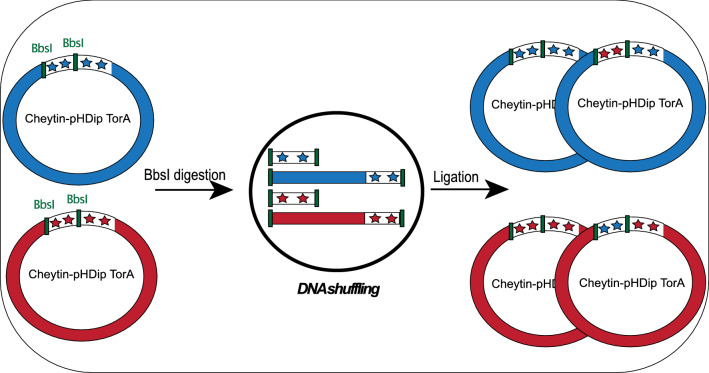
Schematic diagram of the DNA sequence shuffling process for two different Cheytin clones: Example of two phagemids from the library (clone 1 in blue and clone 2 in red). The four diversified loops windows are respectively represented in blue (phagemid 1) and red (phagemid 2) stars. BbsI restriction sites are represented by green lines. Cleavage of the phagemid by the enzyme BbsI makes it possible to split in two the gene coding for Cheytin variants. The ligation of a mixture of the two phagemids, clone 1 and clone 2, digested by BbsI can generate two additional phagemids resulting from the recombination of clone 1/clone 2.

The Lib-Cheytins 2.0 filtrated sub-population was recombined using 2S restriction sites previously introduced in the sequence design. Plasmids from the Cheytins 2.0 pool were digested by BbsI, self-ligated in the same sample (Fig. 4; see “Material and Methods”) and electroporated. The resulting library, Lib-Cheytins 2.1, contained 2.8 × 10^8^ independent clones. Fifteen randomly picked individual clones were sequenced. The fraction of in-frame sequences in the shuffled library, 87% (13/15), is nearly the same as the one obtained in the filtered library Lib-Cheytins 2.0.

### Characterization of the final library

#### Sequence variability in degenerated positions

**Figure 5:**
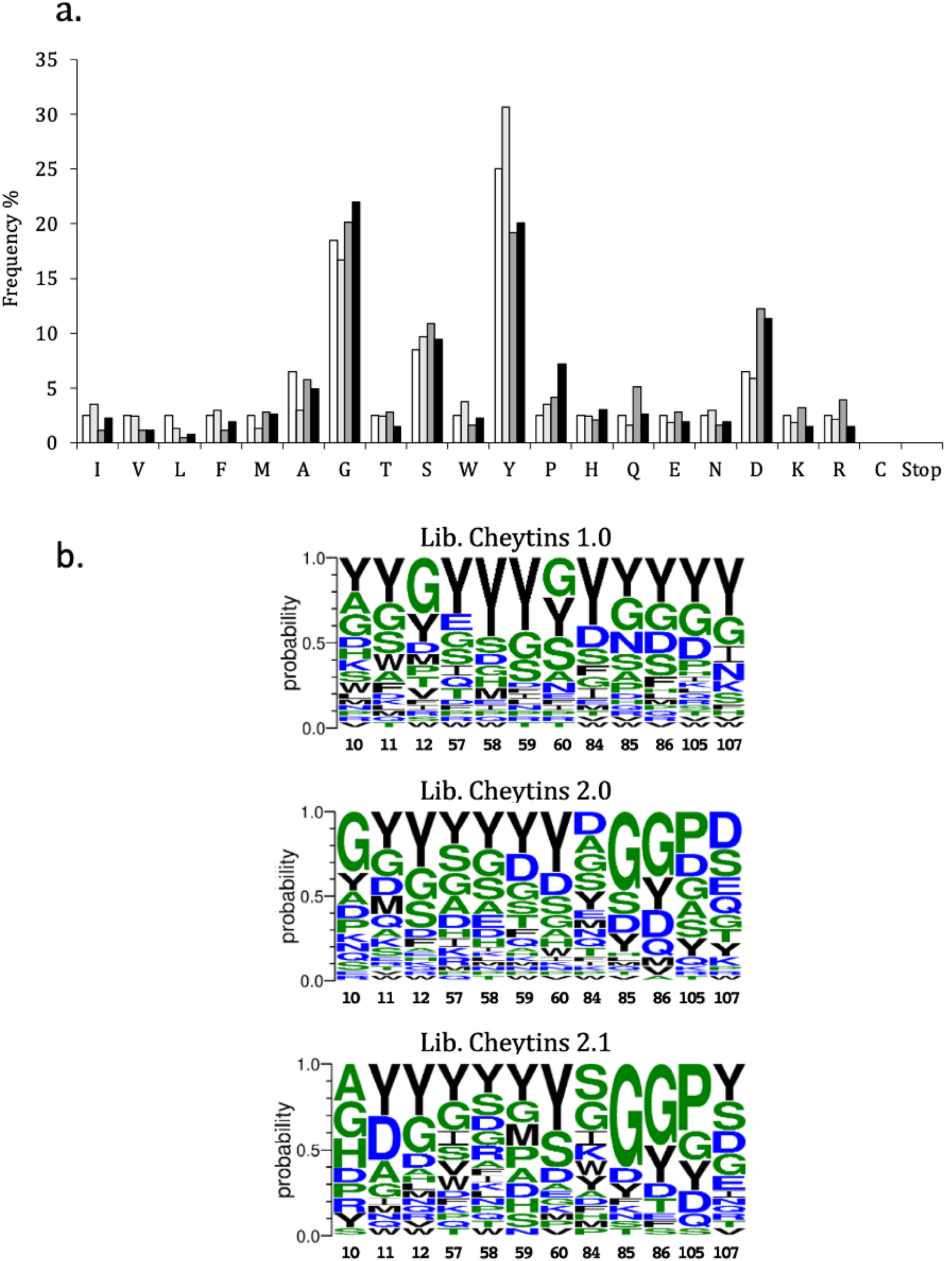
Sequence analyses of the 3 Cheytins libraries 1.0, 2.0 and 2.1. (**a**) Sequence diversity of the three libraries Lib-Cheytins 1.0 (light grey), Lib-Cheytins 2.0 filtrated by P2 (middle grey) and Lib-Cheytins 2.1 (Black) compared to the expected diversity (white). The experimental amino acids frequency of the randomized positions compared to the expected frequencies from the coding scheme (white). (**b**) Logo representation of the 12 randomized positions in the three libraries. The alignments of individual sequences are shown as a sequence LOGO using the sequence numbering found for randomized positions in each library as the abscissa. The sequence diversity (**a**) and the alignments (**b**) were performed with randomly selected protein sequences from the 3 different libraries. Thirty different protein sequences (corresponding to 12 * 30 = 372 diversified amino acid) encoding variants of Lib-Cheytins 1.0, thirty-six different protein sequences for Lib-Cheytins 2.0 (432 diversified amino acid), and twenty-two different protein sequences (264 diversified amino acid) for Lib-Cheytins 2.1 were used for these analyses. The frequency was calculated by considering the number of a particular amino acid found in the totality of the diversified amino acids in the set of sequences of each library. For example, for library 1.0, thirty sequences were considered for this study. In each sequence, there are twelve randomized residues, corresponding in total to 12 * 30 = 372 amino acids. In this sample, we found thirteen prolines. The frequency of proline corresponds to (13/372) * 100 = 3.49%.

Comparisons of the amino acid distribution in the different Cheytins libraries are presented in histograms (Fig. 5a) and LOGOs (Fig. 5b). Histogram analysis of Lib-Cheytins 1.0 shows that the 12 randomized positions were diversified as expected. However, some relative deviations are observed in the filtered (Lib-Cheytins 2.0) (over-representation of aspartate) and in the final (Lib-Cheytins 2.1) libraries (over-representation of aspartate and proline) (Fig. 5a).

Analyses of the LOGO allowed to highlight deviations in the individual randomized positions with an over-representation of glycine in positions 85 and 86 and proline in position 105 in the final library compared to the initial one (Fig. 5b).

#### Characterization of protein stability

We developed a new approach to test the average stability of a pool of proteins selected from each library. A series of clones from each library (11 from the 2.1 library and 12 from the 1.0 library) were chosen on the basis of their soluble expression controlled on SDS-PAGE, and produced as two pools of proteins (Fig. S4).

**Figure 6:**
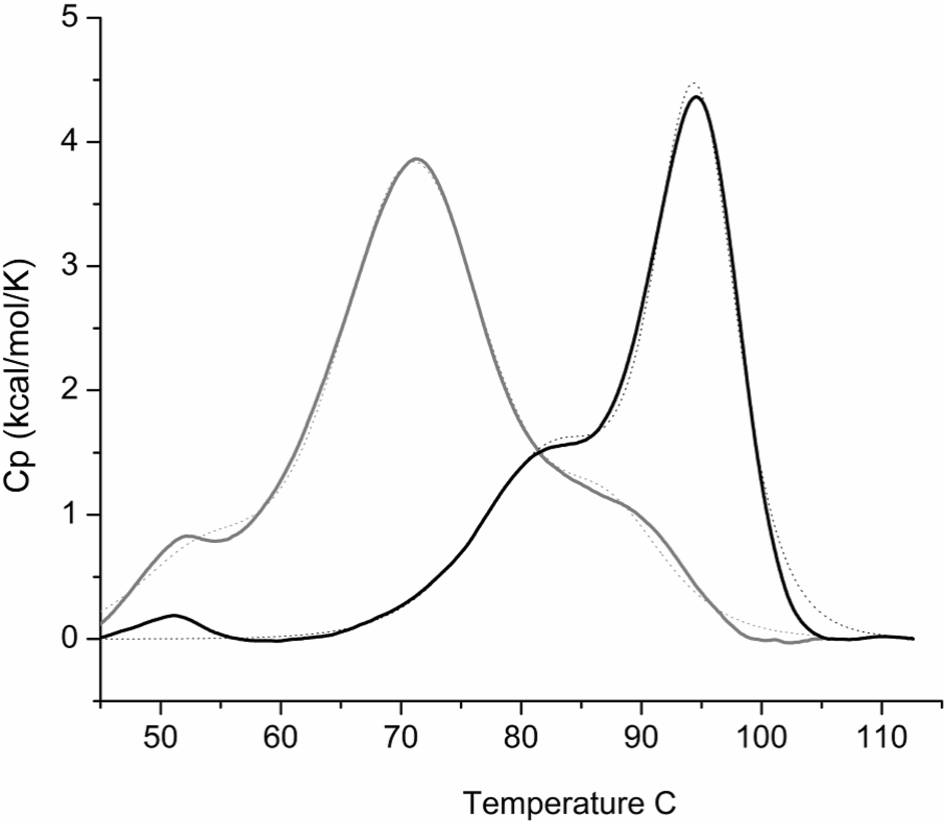
Comparison of the protein stability of variants from Lib-Cheytins 1.0 and Lib-Cheytins 2.1. Heat denaturation of pools of proteins from Lib-Cheytins 1.0 (grey line) and Lib-Cheytins 2.1 (black line) assessed by differential scanning calorimetry (DSC): each pool was purified from a collective production of 11 clones for Lib-Cheytins 2.1 and 12 clones from Lib-Cheytins 1.0 in *E.Coli* Rosetta (DE3) pLysS. Each pool of proteins was loaded at 1.7 mg mL^−1^. All the clones of each pool have been controlled by SDS-PAGE for their soluble expression in *E. coli* (see supplementary data Fig. S4).

In this experiment, a mixture of plasmids with equal concentrations of plasmids coding for each protein was used for the *E. coli* transformation. Expression and purification of this pool of plasmids was performed as described in Materials and Methods section. The stability of each pool of proteins was measured by DSC using samples adjusted at the same protein concentration (1.7 mg mL^−1^). Figure 6 shows a significant shift between the 2 thermograms. The pool of library 2.1 proteins displays a distribution of unfolding transitions centred around 95 °C whereas the thermogram of the library 1.0 pool of proteins is centred around 71 °C. Mass spectrometry experiments were performed to identify Cheytin variants present in the 2 pools (Table S3). Almost all proteins were present as: 10/12 and 11/11 proteins were respectively identified in Cheytin 1.0 and Cheytin 2.1 pools.

Individual clones from the 2 libraries were also separately purified and their DSC profiles were recorded (Fig. S5). The 3 clones from the library 2.1 are more stable (average Tm = 88.3 ± 4.9 °C) than the 3 clones from the library 1.0 (average Tm = 73.7 ± 1.08 °C).

These results show that proteins of the Lib-Cheytins 2.1 either monitored individually or collectively are more stable than proteins from the primary Lib-Cheytins 1.0. This validates the new “conformational filtration” approach using P2, as an efficient approach to increase stability of the library variants.

### Selection for specific Cheytin binders

At this step the main goal was to test if the final library, Lib-Cheytins 2.1, could efficiently give rise to Cheytin-variants with specific binding properties for pre-defined targets. The Kaz luciferase-derived protein was chosen as a target. Due to its small size and high activity Kaz luciferase, and related proteins, have found a range of applications as light emitting tracers. Our aim was to develop a protein module that could be used as an intramolecular inhibitor of Kaz luciferase for a further incorporation in allosteric biosensors.

The Kaz derived from the 19 kDa fragment of the *Oplophorus* luciferase (AB823628) is prone to aggregation when produced in *E. coli* but was however reported to be well-expressed and active as a fusion with a solubilizing partner (ZZ domain)^48^. To efficiently produce this Kaz subunit, the Kaz sequence was fused to a stable, highly soluble artificial protein based on a previously described alphaRep^20,49^. Here the alphaRep acts as a solubilizing partner like the ZZ domain and has no specific binding site for the Kaz protein. The resulting protein the “Kazα” was highly produced (20 mg for 1 L of culture), then purified in a well folded, soluble and biotinylated manner when fused to a cleavable biotinylation tag (Avi-tag).

#### Selection from the Lib-Cheytins 2.1 of Kaz binders

**Figure 7:**
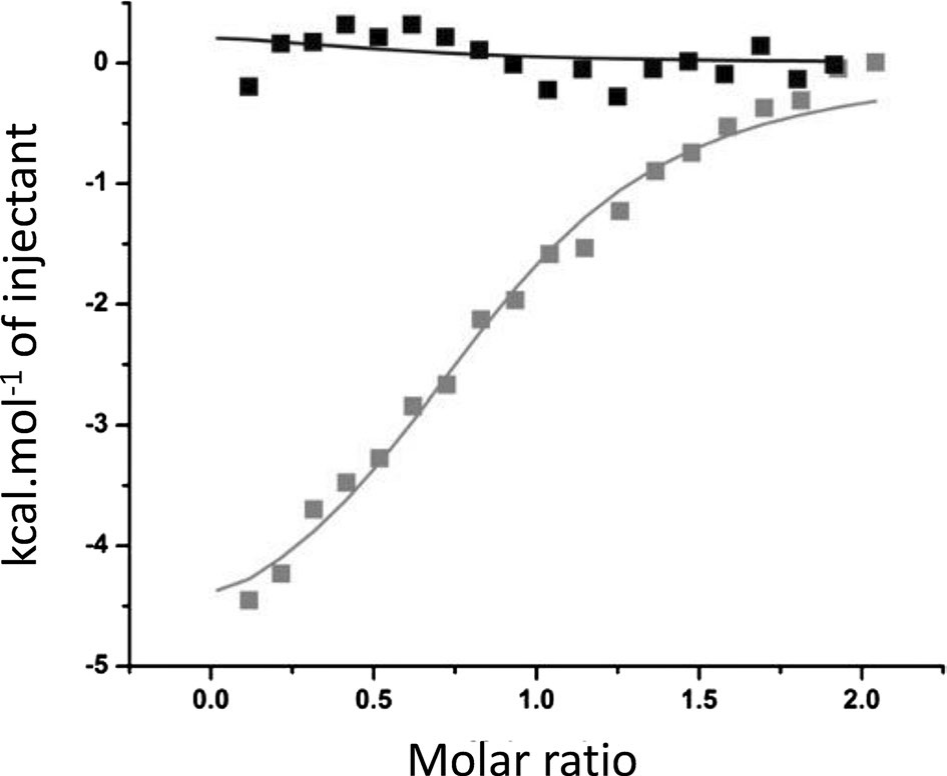
Interactions of the bKF5 with the Kazα target assess by ITC. In Grey: Isothermal calorimetric titrations of Kazα (25 μM) with the bKF5 binder (220 μM). In Black: calorimetric titrations of Kazα (25 μM) with CheytinWT (220 μM). Parameters of the binding reaction, K_D_ and stoichiometry (n) are extrapolated from the saturation curve.

Briefly, three rounds of selection were performed with the Lib-Cheytins 2.1, using biotinylated Kazα bound to streptavidin on an ELISA Plate. To prevent the selection of Cheytin binders interacting with the alphaRep solubilizing moiety, bound phages were specifically eluted by releasing the immobilized Kaz-protein from the AlphaRep fusion with TEV protease. The Kaz-binding clones were identified using a clonal phage-ELISA screening step (Fig. S6). The bacteriophages produced from individual Cheytin clones were incubated in presence of the immobilized target and detected using an anti-phage antibody. Several Cheytin-variants identified as positive in this assay were sequenced and sub-cloned into a cytoplasmic expression vector (pQE81L). The individual variants, named bK (for **b**inder of **K**az), were highly produced with a yield of 10 to 27 mg per litter of culture (bKF5 = 18 mg; bKG10 = 27 mg; bKE4 = 20 mg; bKD5 = 14.4 mg; bKA11 = 9.9 mg per liter of *E.coli* culture). The interactions between the Kazα target and its binders were measured by ITC and the dissociation constant (K_D_) values were determined (Fig. S7) and found in the μM range. ITC titration between the selected Kaz binder bKF5 and the Kazα target shows a K_D_ of 3.9 ± 0.7 µM and a stoichiometry of 0.85 ± 0.03 whereas, as expected, no interaction was observed for the non-selected CheytinWT (Fig. 7). Moreover, no interaction was observed between the bKF5 and Lyzozyme, showing bF5Kaz does not interact non-specifically with proteins not related to its cognate target (Fig. S8).

#### Inhibition of Kaz luciferase activity by the selected Cheytin binders

**Figure 8:**
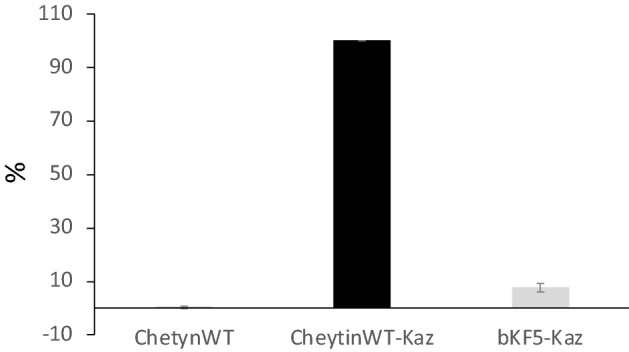
Luminescence activity of the CheytinWT (dark-grey), of the fusion CheytinWT-Kazα (black), and the bKF5-Kazα (low-grey) after addition of the Nano-Glo substrate. For each experiment, the maximal luminescence activity obtained with the CheytinWT-Kazα has been defined as the 100% activity and all other values (CheytinWT, and bKF5-Kazα) have been normalized accordingly. For each protein sample, 3 independent experiments were performed. For each experiment, the average of triplicate measures is calculated. Each measure corresponds to the average of 20 records of the Kaz activity luminescence signal monitored during 1 min in initial rate conditions.

Kaz luminescence activity is highly sensitive and can only be measured at very low enzyme concentrations. In these conditions, a stochiometric ratio of bKF5 and Kaz would be well below the dissociation constant of the complex. As previously described, interactions between a protein binder and its target can be strengthened by the addition of a peptide link between the two domains, inducing intramolecular interactions^50^. Therefore, to test if an enzyme inhibition would result from a stochiometric binding of bKF5, the enzyme (Kazα) was fused to its binder (bKF5) through a peptide linker. The resulting fusion protein was named bKF5-Kazα. As a control, an equivalent fusion protein CheytinWT-Kazα was designed from the non-relevant CheytinWT protein and the Kaz-alphaRep. The two Kaz protein-fusions were tested for their luminescence activity by addition of a Kaz substrate (NanoGlo^**®**^) (Fig. 8). For the bKF5-Kazα fusion, 92% of the luminescence activity was lost compared to the CheytinWT-Kazα activity. This suggests that the bKF5 selected against Kaz could indeed interact with the Kaz active site and consequently inhibit the luminescence activity of the enzyme.

## Discussion

CheY protein of *Thermotoga maritima* was retained as a starting scaffold due to its high initial stability and to detailed available knowledge on stabilizing mutations. Additionally, the response regulator fold is found in many different two component signaling pathways presumably due to its intrinsic propensity to adapt to a range of protein partners. In these processes, CheY protein surfaces are involved in interactions with different partners showing its structural plasticity. This suggests that its surface could potentially adapt to a range of sequences and could be favorable to fit to different protein partners. Finally, the structure of CheY bound to a P2 domain of CheA shows that this interaction could be used to probe the structural integrity of proteins variants.

The initial library of degenerated genes coding for individual Cheytin sequences were synthesized by circular amplification of self-assembled oligonucleotides. Amplification based on RCA is highly efficient but only with circular sequence. Using this approach, partially assembled oligonucleotides resulting in non-circular sequences are not amplified^20^.

In Lib-Cheytins 1.0, the sidechains distribution in variable positions was evaluated experimentally and corresponded to the diversity expected from the coding scheme, with no clear diversity bias. This analysis demonstrates that trinucleotide randomization is efficient to result in the expected representation of codons in each randomized position.

Selecting folded proteins from a highly randomized sequence collection is a demanding task and there is no easy process to screen for folded sequences^51,52^. Several hypotheses were tested to improve the well-folded proteins library quality.

First, by evaluating the effect of the different secretory pathways, we showed an improvement of the display of CheY variants by the TAT pathway during the phage display selections comparing with the Sec pathway. Since the TAT system allows secretion of folded proteins from the *E. coli* cytosol^45^, it might take advantage over the Sec system, in particular, for those proteins, as CheY, which are highly stable and that can possibly fold fast in cytoplasm before translocation^53^. This secretion system may also contribute to the optimization of the phage-display selection by displaying well-folded proteins^45,53^.

Secondly, we successfully used the P2 protein, a biological partner of CheY, to specifically capture correctly folded variants of the Lib-Cheytins 1.0 library exposed at the surface of a phage.

This approach allowed to recover 2.8 × 10^6^ independent clones in Lib-Cheytins 2.0, with a high proportion 90% (2.5 × 10^6^) of the expected coding sequences. This represents 5.7% of the correct sequences from Lib-Cheytins 1.0. The loss of diversity during this capture process could be explained either by a sub-optimal capture of the phages displaying a folded protein during the filtration process or by a small fraction of correctly folded sequences in interaction with P2 within the initial library. To compensate the diversity loss inherent to the filtration step and increase the diversity of the final library, these filtered coding sequences were then shuffled using a one-step simple and efficient procedure and a new library Lib-Cheytins 2.1 composed of 2.8 × 10^8^ independent clones with 87% correct coding sequences was successfully obtained. The shuffling process of a set of coding sequences is an efficient way to increase the diversity of the library while keeping a low proportion of frame shifted or out of frame sequences.

Interestingly, we observed deviations in the amino acid frequencies in the experimental filtrated libraries as compared to the expected diversity. An over-representation of the aspartate is observed in the filtered library and an over-representation of the aspartate and proline is observed in the final library. By analysing LOGO corresponding to the different libraries, the deviations observed do not affect all the positions but only specific positions. Indeed, deviations are observed in limited individual randomized positions with an over-representation of glycine in positions 85 and 86 (in the libraries, 2.0 or 2.1) and proline in position 105 in the final library compared to the initial 1.0 library. Remarkably, these residues are positioned in the nearest loops close to the area interacting with P2, and two of these residues in positions 85 and 105 are present in the wild type CheY protein (Gly85 and Pro105). The origin of these biases may result from a structural constraint as a consequence of the filtration upon P2 binding. This analysis of a small population of clones reveals only the most visible differences between the filtered and unfiltered library. The analysis of a larger sample of these populations by NGS could possibly allow more detailed analysis.

The interactions between P2 and CheY correspond to the outcome of a natural evolution process. By using P2 as a filtration tool, we probably re-introduced drift mimicking this natural evolution and we favored the selection of variants presenting the specific features essential for the interaction with P2.

To validate the conformational filtration, we have shown that four randomly picked variants issued from the filtrated library 2.0 are still able to bind P2 (Fig. S3). This demonstrates that the randomization process does not affect the ability of the variants to bind the P2 partner and corroborate our assumption that a filtration with P2 is possible to capture an ensemble of well-folded variants.

Importantly the results show that the selected sequences in Lib-Cheytins 2.0 are as expected properly folded and stable proteins but moreover that these favorable properties are preserved upon recombination. Indeed, the distribution of unfolding transitions as assessed by DSC is increased by 24 °C in the 2.1 pool of proteins compared to the 1.0 pool. Analysis of randomly selected proteins from each library confirms this observation: proteins from the Lib-Cheytins 2.1 are on average, clearly more stable than proteins from the primary Lib-Cheytins 1.0. Randomization inevitably introduces highly destabilizing substitutions in the randomized sequence population, for example due to steric clash within the scaffold, or local sequence/structure incompatibilities. A strong local destabilization within a protein coding sequence often has a dominant effect as the whole protein is no longer able to fold whatever the sequences of other randomized loops. The filtration step based on the interaction with protein partner eliminates most of fold-incompatible local sequences. Furthermore, the filtration step was conducted with proteins displayed on phage surfaces which have been efficiently secreted through the TAT dependent export pathway. This export pathway has been described to selectively export^54,55^ folded proteins. The selected population from Lib-Cheytins 2.0 contains protein variants that cumulate two criteria for foldability: efficient display through the TAT export pathway and conformational dependent recognition by a protein partner.

Consequently, the new pool of sequences generated by recombination contains not only randomized but also foldable sequence fragments. The filtration/shuffling process does not remove from the library destabilized proteins resulting from individually stable but mutually incompatible pairs of fragments. The results presented here suggest that, if mutual incompatibilities between pairs of fragments exist, this is not the most frequent cause of destabilization in the pool of randomized sequence. The recombination of viable sequences described above is conceptually similar to the family shuffling procedures which proved efficient to explore a sequence space enriched in viable sequences^56^.

Comparative studies on binders selected from different scaffolds suggest that high affinity binders usually bind their cognate targets using buried surface areas from 700 to 1200 A^2^, which is potentially obtainable if most of the 12 randomized residues in Cheytin are involved in the binding interactions^42^. The affinity of the selected binders against the KAZ domain was lower (µM range) than the binding force generally obtained by some synthetic antibody or other scaffold libraries for example DARPins^19^ or alphaReps^20^ (sub nM to nM range). This may be related to the lower number of variable positions in the diversified loops and thus the diversity of potential binding surfaces. For applications requiring high affinity binders, subsequent affinity maturation steps could potentially be useful.

The topology of the binding surface has also been investigated^57^, while relatively flat surfaces are typically bound with repeat proteins such as DARPins or alphaReps. The protruding loops of VHH or related architectures such as monobodies/Fn3 libraries have given rise to binders that fits in concave areas such as enzyme active sites although nothing in the selection procedures explicitly orientate the putative binders towards a specific area of the enzyme surface. The topology of the scaffold surface may therefore be of importance to select enzyme inhibitors. The randomized surface of Cheytins is also composed of flexible loops and for this reason we used this library to look for inhibitors of Kaz activity. Without exhaustive screening, we rapidly found an enzyme inhibitor among the selected binders. Our goal is now to design biosensors based on Kaz binders capable of modulating luminescence activity for further developments in diagnosis or detection.

## Materials and methods

Information concerning the nucleotide sequences coding for the different proteins studied here are deposited on GenBank. The accession numbers corresponding to these sequences are presented in Table S1.

### Library acceptor vectors

A phage display acceptor vector (pHDip Tor A-acc) was used for the library construction. This vector has a filamentous phage M13 replication origin, a bacterial replication origin, a gene coding for the Betalactamase to confer ampicillin resistance. The library proteins are expressed in fusion with, upstream, a Tor A export sequence and, downstream, the C-terminal domain of the pIII protein of the filamentous phage M13. Sub-cloning of the variant genes was carried out using two BsaI restriction sites. A KpnI site is also present between the two BsaI sites (Fig. 2b).

### Degenerated oligonucleotides

Sequence of the oligonucleotides used for the Cheytin library synthesis, produced by Ella biotech (https://www.ellabiotech.com/production/trim), are presented below. Positions submitted to randomization using the trimer phosphoramidites are indicated by a cross. The mixtures of trinucleotides were settled according to the frequencies listed in supplementary Table S2.

1. 5′-attaccaaagcgggctatgaagtcgcaggcgaagctaccaacggt-3′
2. 5′-cgtgaagccgtcgaaaaatactatgaactgaaaccggatatc-3′
3. 5′-atcgaagacattatgtatatcgatccgaacgcaaaaatc-3′
4. 5′-gcactgaataaagtctcaaagGGCTaGAGACCaaaaggtctca-3′
5. 5′-GCGAtcGAAGACcgaggcaaacgtgtgctgattgttGATXXXaacatgcgtatgatgctgaaagacatt-3′
6. 5′-gttaccatggacatcacgXXXXaacggcattcgtgct-3′
7. 5′-atcgtgatgagcgcgXXXcaggccatggttattgaagcaatcaaa-3′
8. 5′-gcgggtgccaaagacttcattgtcaaaXttcXccgagccgtgttgtcgaa-3′
9. 5′-gacttcatagcccgctttggtaataatgtctttcagcatcatacgcatgtt-3′
10. 5′-tttttcgacggcttcacgaccgttagcttcgcctgc-3′
11. 5′-cgtgatgtccatggtaacgatatccggtttcagttcatagta-3′
12. 5′-gatatacataatgtcttcgatagcacgaatgccgtt-3′
13. 5′-cgcgctcatcacgatgatttttgcgttcggatc-3′
14. 5′-tttgacaatgaagtctttggcacccgctttgattgcttcaataaccatggcctg-3′
15. 5′-ctttgagactttattcagtgcttcgacaacacggctcgg-3′
16. 5′-aacaatcagcacacgtttgcctcggtcttcgatcgctgagaccttttGGTCTCtAGCC

### Synthesis of the microgenes coding the Cheytin-variants

To construct the first-generation library (Lib-Cheytins 1.0) the full-length gene encoding for Cheytin-variant proteins was created by assembling sixteen synthetic primers containing the random and the constant TRIMers codons from Ella biotech^58^. Four 5′-phosphorylated oligonucleotides Variables (V1–V4) and four Constants (C1–C4) corresponding to the coding strand of the randomized sequences were hybridized with eight reverse oligonucleotides, complementary to these coding strand oligonucleotides. The hybridized oligonucleotides were pre-assembled in 4 Blocks (Block A (Rev − 1 + V1 + C1 + REV 2); Bloc B (C2 + V2 + Rev 2 + Rev 3); Bloc C (C3 + Rev 4 + V3 + Rev 5); and Bloc D (Rev 6 + V4 + Rev 7 + C4)) to minimize incorrect assemblies by incubation 15 min at 65 °C followed by 15 min at 37 °C. Each Block mix was prepared in a total volume of 100 μL in the suitable buffer at 10 µM final oligonucleotide concentration. These blocks were mixed (7 μM each in 0.5 mL final volume), ligated by T4 ligase to give circular products that were used as substrates for Rolling circular amplification (RCA) with Phi29 polymerase (TempliPhi kit, GE Healthcare).

A 7 nmol (1 μL) sample of circularized product was mixed for 18 h at 30 °C in 20 μL of amplification reaction mix in the presence of phi29 polymerase. The polymerized product was incubated at 65 °C for 15 min to inactivate the polymerase, diluted to 100 μl with water and buffer and digested (30 U of BsaI) for 2 h at 50 °C. Agarose gel electrophoresis of the cleaved amplified products showed a 400 bp band as expected from the length of the amplified sequence (394 bp, data not shown). The BsaI digested product was purified and desalted on Nucleospin column and eluted in 50 µL autoclaved H_2_O.

### Library construction

#### Construction of the primary library Lib-Cheytins 1.0

The library Lib-Cheytins 1.0 was constructed by ligation of the synthetic microgenes corresponding to the Cheytin-variants in a phage display acceptor vector (pHDip TorA-acc). The resulting phagemids of the library carry a Lac promoter; the coding sequence corresponds to the Twin-Arginine periplasmic signal sequence (TorA), the sequence of the Cheytin-variant fused to gene IIIp (gIIIp) of M13 (sequence 249–406).

The acceptor vector was first cleaved by BsaI generating linear DNA with two cohesive ends.

For the library construction, 8 µg of linearized vector was ligated with 23 µg of microgenes in 1 mL (vector/microgene molar ratio approximately 1:27), overnight at 16 °C. The ligated product was purified on Nucleospin^Ⓡ^Gel and PCR clean-up (Macherey–Nagel) columns and cleaved by KpnI (NEB) to inactivate the parental acceptor vector (Fig. 2a). The product obtained was then purified, desalted on Nucleospin column and eluted in 50 µL autoclaved H_2_O.

DNA was electroporated in XL1-Bl’e MRF' electroporation supercompetent cells (Agilent technology) using the MicroPulser™ (Bio-Rad) with standard conditions (22.5 kV/cm, 200 Ω, 25 μF). Ten 50 µL samples of electrocompetent cells were electroporated with 400 ng of final ligation, and plated on 24.5 × 24.5 cm agar plates in 2YT medium containing 200 µg mL^−1^ ampicillin and 1% (w/v) glucose. Dilutions of the electroporated cells were plated separately to evaluate the size of the library. Colonies were harvested after overnight growth, pooled and stored at − 80 °C in 2YT medium, 20% (v/v) glycerol.

#### Construction of the filtrated Lib-Cheytins 2.0

For the construction of the second-generation library (Lib-Cheytins 2.0), a solution of phages produced from Lib-Cheytins 1.0 (10^13^ phages) was incubated on an ELISA plate coated with biotinylated P2 protein (40 μg mL^−1^) immobilized on streptavidin (20 μg mL^−1^) and blocked with a solution of TBS (20 mM Tris–HCl pH 8.0, 150 mM NaCl) containing BSA (3% W/V) and Tween-20 (0.1% V/V) (TBST-BSA). Proteins were exposed on the phages via a C-terminal fusion with the M13-PIII. Retained phages displaying a correct folding may enabled the interaction with the P2 partner. The ELISA plates were washed and the captured phages were eluted by a specific TEV protease digestion (10 µg mL^−1^), overnight at 4 °C. Freshly prepared bacteria were infected by the recovered phages and plated. Plasmids were recovered as a pool from this ‘‘filtrated’’ bacteria population using Macherey–Nagel DNA extraction Kit. The Cheytin-sequences from this plasmid pool obtained by Bsa1 digestion were extracted from agarose gel, circularized by self-ligation and reamplified using RCA.

This population of recovered DNA fragments was ligated into BsaI digested plasmids pHDip TorA-acc. The ligated products were transformed into XL1-Blue MRF’ electro-competent cells as described for the first-generation library and the final cell suspension stored as glycerol stocks of the Lib-Cheytins 2.0.

#### Construction of the filtrated-shuffled Lib-Cheytins 2.1

To increase the diversity of the second-generation library, the purified DNA population was shuffled generating new combinations of DNA sequences. Exactly, 6 μg of DNA was digested by BbsI (NEB) and after enzyme deactivation at 80 °C, re-ligated directly in the same sample overnight at 16 °C (Fig. 4). The ligated products were electroporated in XL1-Ble MRF' electroporation competent cells (Agilent technology); colonies were harvested from the plates, pooled and stored at − 80 °C as described for the Lib-Cheytin 1.0. This constitutes the final library Lib-Cheytins 2.1.

To further analyze the different libraries, a sample of pooled bacteria was used to prepare a DNA pool for restriction analysis. Sub-cloning of the gene variants in a protein expression vector and a collective transformation in a high-level expression host was then carried out.

### Monitoring of soluble expression

For cytoplasmic protein expression, the DNA fragment containing only the coding sequence of a protein without export sequence was sub-cloned into the PQE80L expression vector by a Gibson assembly approach. The genes coding for a set of protein variants with complete correct coding sequences (without frameshift or stop codon) were chosen in library Lib-Cheytins 1.0 and 2.1.

The corresponding plasmids were transformed into the *E. coli* expression strain Rosetta (DE3) pLysS. The culture conditions and the sample preparations were carefully and reproducibly calibrated.

Cells were grown at 37 °C in 2YT medium containing 200 µg mL^−1^ ampicillin to an absorbance of 0.6 at 600 nm. Protein expression was induced by addition of 1 mM IPTG and the cells were further incubated for 4 h. The bacterial sample were suspended at a final OD_600_ = 4, in a lysis buffer (B-PER Thermo Fisher Scientific) supplemented with DNase 1 (1 U/mL; Thermo-Fisher) incubated 30 min at 20 °C. These samples corresponded to the bacterial total extract (TE). The soluble fractions (SF) were obtained by centrifugation of the TE samples for 30 min 14,000 g 4 °C.

SDS-PAGE (15% acrylamide) was then performed dispensing alternatively 15 μL of TE and SF samples for each clone.

### Protein expression and purification

#### Cheytin-variant- pool or individual clones

The *E. coli* strains M15 (pREP4) or Rosetta pLysS (Qiagen) were transformed by the plasmid (or mixture of plasmids for the pool) coding for the Cheytin-variants. Cells were grown at 37 °C in 2YT medium containing 200 µg mL^−1^ ampicillin until the absorbance OD 600 nm reached 0.6. Protein expression was induced by addition of IPTG (0.5 mM final concentration). Cells were incubated 4 h at 37 °C. Then they were harvested, suspended in TBS supplement with anti-protease (PIC Roche), submitted to two freezing/thawing cycles, treated with DNase 1 and sonicated.

The His_6_-tagged proteins were all purified from crude supernatant using nickel-affinity chromatography (Ni–NTA agarose, Qiagen) followed by size-exclusion chromatography (Hiload 16/60 SuperdexTM 75) in Cheytin-buffer.

#### Natural Cheytin binder: P2 domain CheA

The synthetic gene encoding the P2 domain of the histidine Kinase CheA was purchased from IDT and cloned in a PQE81L vector. The P2 coding-sequence was separated by a TEV protease cleavage site sequence from an AviTag sequence introduce in the 3′ end of the gene. The resulting protein was composed of His_6_-P2-TEV-AviTag.

#### Protein target for Phage display selection

The synthetic gene coding for the fusion of the Kaz with the AlphaRep, used as a target for the selection of binders, was cloned in a PQE81L vector. Both sequences were separated by a TEV protease site sequence and an AviTag™ sequence was also introduce in the 3′ end of the gene. The resulting protein named “Kα” was composed of His_6_-Kaz-TEV-AlphaRep-AviTag.

For both proteins, P2 and Kα, plasmids were transformed into BL21 cells previously transformed with pBirAcm (Avidity) allowing IPTG inducible biotin ligase expression^59^. Cytoplasmic expression and biotinylation of the proteins were induced as described in^49^. These fusion proteins produced at high level in *E. Coli* were purified using nickel-affinity chromatography followed by size-exclusion chromatography (Hiload 16/60 SuperdexTM 75) as described above.

#### Fusion proteins with Cheytin-variants

- Two different genes encoding fusion proteins were constructed to test the specificity of Cheytin binders selected against Kaz-AlphaRep. First the CheytinWT-Kaz-alphaRep fusion protein (the CheytinWT-Kα), corresponds to the fusion between the CheytinWT and the Kα. Second the fusion bF5K-Kα, corresponds to the fusion between the binder F5 of Kaz, selected from the Lib-Cheytins 2.1 and the Kα. These two genes cloned in PQE81L-vector were obtained by the Gibson assembly approach (data not shown). CheytinWT-Kα and bF5K-Kα were purified using an affinity chromatography (Histrap™ FF crude 5 mL GE Healthcare); samples obtained from this first step purification were then submitted to a size-exclusion chromatography (Hiload 16/60 SuperdexTM 75) equilibrated in Cheytin-buffer Tris 20 mM NaCl 150 mM MgCl_2_ 5 mM pH 7,4.

The purity of the final sample corresponding to each protein was controlled by SDS–PAGE. Protein concentrations, expressed as monomers, were quantified by UV spectrophotometry.

### Differential scanning calorimetry

Thermal stability of the Cheytin-variants was performed by DSC on a MicroCal VP-Capillary DSC Calorimeter from Malvern in a standard buffer. Each measurement was preceded by a baseline scan with the standard buffer. Scans were performed at 1 K min^−1^ between 20 and 110 °C. The heat capacity of the buffer was subtracted from that of the protein sample before analysis. Thermodynamic parameters were determined by fitting the data with the following equation:

$$\Delta C_{p} (T) = \frac{{K_{d} (T)\Delta H_{cal} \Delta H_{vH} }}{{\left[ {1 + K_{d} (T)} \right]^{2} RT^{2} }}$$

where K_D_ is the equilibrium constant for a two-state process, ΔH_vH_ is the enthalpy calculated on the basis of a two-state process and ΔH_cal_ is the measured enthalpy.

### Isothermal titration calorimetry

The binding parameters were measured with an ITC 200 microcalorimeter (MicoCal, Malvern) at 25 °C.

2 μL aliquots of the titrant were injected from a computer-controlled 40 μL microsyringe at intervals of 180 s into the solution of target dissolved in relevant buffer (stirring at 800 rpm).

The data were integrated and analyzed using the MicroCal Origin software provided by the manufacturer according to the one-binding-site model.

### Phage display procedure

The final library 2.1 was used for the selection of binders against the Kaz protein. Phages were prepared from the library Lib-Cheytins 2.1 as described in^20^. Briefly XL1-Blue MRF’ bacteria corresponding to the phagemid libraries were infected with the helper phage (M13KO1)^60^. After replication of phages overnight at 30 °C, the cultures were centrifuged at 5000 g for 30 min. The phage-containing supernatant was dialyzed against the Tris-buffered saline (TBS-20 mM Tris/HCl, pH 8.0, 150 mM NaCl), using a 300 kDa MWCO dialysis membrane to remove free proteins from the phage solution.

#### Selection of Cheytin-variants binders against Kaz

Selections with the final library Lib-Cheytins 2.1 were performed as described in^49^ except for the following modifications. The in vivo biotinylated Kα was linked on streptavidin coated micro-titre ELISA plate. To prevent the selection of streptavidin-binding clones, phages from the library were pre-incubated in wells coated with streptavidin (1–2 × 10^10^ phages/well) and then transferred to the selection plate for 1 h at 20 °C. After several washes with Tris-buffered saline and Tween 20, bound phages were specifically eluted by releasing the immobilized Kaz-protein with TEV protease (10 μg mL^−1^) for 3 h at 25 °C. After three rounds of selection, specific clones were identified by Phage-ELISA screening as previously described^49^.

### Monitoring of Kaz luminescent activity

Luminescence activity was measured on a Tecan Infinite 200 PRO plate reader or on a Clario-star plus plate reader, using a 96-well, black, flat bottom (Material number: 30122298). All measurements were done in a 300 μL final volume, in buffer B (Tris–HCl 20 mM NaCl 150 mM MgCl_2_ 5 mM -pH7,4) supplemented with 0,1% BSA.

The Cheytin-WT-Kaz-alphaRep protein fusion (WT-K) and the binder Cheytin-bKaz/ Kaz-alphaRep protein fusion (bF5K-K) activities were monitored using a Kinetic mode by quantifying the emitted luminescence during 1 min. The experimental conditions were chosen to measure luminescence activity in initial rate conditions. For each sample, the final concentration of protein was 0.01 nM; the Nano-Glo^®^ (Nano-Glo^®^ Luciferase Assay System, Promega), substrate of the Kaz, was added at final concentration corresponding to 3000 ppm, the samples were shaken two seconds before measurement.

## Supplementary Information

Supplementary Information 1.

Supplementary Information 2.

## Data Availability

Information concerning the nucleotide sequences coding for the different proteins studied here are deposited on GenBank. The accession numbers corresponding to these sequences are presented in Table S1.

## References

[1] Tiller KE, Tessier PM (2015). Advances in antibody design. Annu. Rev. Biomed. Eng..

[2] Shepard HM, Phillips GL, Thanos CD, Feldmann M (2017). Developments in therapy with monoclonal antibodies and related proteins. Clin. Med. (Lond.).

[3] Moutel S (2016). NaLi-H1: A universal synthetic library of humanized nanobodies providing highly functional antibodies and intrabodies. Elife.

[4] Jost C, Pluckthun A (2014). Engineered proteins with desired specificity: DARPins, other alternative scaffolds and bispecific IgGs. Curr. Opin. Struct. Biol..

[5] Azhar A (2017). Recent advances in the development of novel protein scaffolds based therapeutics. Int. J. Biol. Macromol..

[6] Gebauer M, Skerra A (2019). Engineering of binding functions into proteins. Curr. Opin. Biotechnol..

[7] Koide S (2009). Engineering of recombinant crystallization chaperones. Curr. Opin. Struct. Biol..

[8] Mittl PR, Ernst P, Plückthun A (2020). Chaperone-assisted structure elucidation with DARPins. Curr. Opin. Struct. Biol..

[9] Bieli D (2016). Development and application of functionalized protein binders in multicellular organisms. Int. Rev. Cell Mol. Biol..

[10] Harmansa S, Affolter M (2018). Protein binders and their applications in developmental biology. Development.

[11] Rinne SS, Orlova A, Tolmachev V (2021). PET and SPECT imaging of the EGFR family (RTK class I) in oncology. Int. J. Mol. Sci..

[12] Owens B (2017). Faster, deeper, smaller-the rise of antibody-like scaffolds. Nat. Biotechnol..

[13] Gebauer M, Skerra A (2020). Engineered protein scaffolds as next-generation therapeutics. Annu. Rev. Pharmacol. Toxicol..

[14] Lee SC (2012). Design of a binding scaffold based on variable lymphocyte receptors of jawless vertebrates by module engineering. Proc. Natl. Acad. Sci. U.S.A..

[15] Taverna DM, Goldstein RA (2002). Why are proteins marginally stable?. Proteins.

[16] Zeldovich KB, Chen P, Shakhnovich EI (2007). Protein stability imposes limits on organism complexity and speed of molecular evolution. Proc. Natl. Acad. Sci. U.S.A..

[17] Gronwall C, Stahl S (2009). Engineered affinity proteins–generation and applications. J. Biotechnol..

[18] Smith GP (2019). Phage display: Simple evolution in a petri dish (Nobel lecture). Angew. Chem. (Int. ed. Engl.).

[19] Binz HK, Stumpp MT, Forrer P, Amstutz P, Plückthun A (2003). Designing repeat proteins: Well-expressed, soluble and stable proteins from combinatorial libraries of consensus ankyrin repeat proteins. J. Mol. Biol..

[20] Urvoas A (2010). Design, production and molecular structure of a new family of artificial alpha-helicoidal repeat proteins (alphaRep) based on thermostable HEAT-like repeats. J. Mol. Biol..

[21] Koide A, Wojcik J, Gilbreth RN, Hoey RJ, Koide S (2012). Teaching an old scaffold new tricks: Monobodies constructed using alternative surfaces of the FN3 scaffold. J. Mol. Biol..

[22] Koide A, Gilbreth RN, Esaki K, Tereshko V, Koide S (2007). High-affinity single-domain binding proteins with a binary-code interface. Proc. Natl. Acad. Sci. U.S.A..

[23] Woldring DR, Holec PV, Zhou H, Hackel BJ (2015). High-throughput ligand discovery reveals a sitewise gradient of diversity in broadly evolved hydrophilic fibronectin domains. PLoS ONE.

[24] Woldring DR, Holec PV, Stern LA, Du Y, Hackel BJ (2017). A gradient of sitewise diversity promotes evolutionary fitness for binder discovery in a three-helix bundle protein scaffold. Biochemistry.

[25] Chevalier A (2017). Massively parallel de novo protein design for targeted therapeutics. Nature.

[26] Golinski AW, Holec PV, Mischler KM, Hackel BJ (2019). Biophysical characterization platform informs protein scaffold evolvability. ACS Combin. Sci..

[27] Léger C (2019). Ligand-induced conformational switch in an artificial bidomain protein scaffold. Sci. Rep..

[28] Léger C (2020). Picomolar biosensing and conformational analysis using artificial bidomain Proteins and terbium-to-quantum dot Förster resonance energy transfer. ACS Nano.

[29] Wolanin PM, Thomason PA, Stock JB (2002). Histidine protein kinases: Key signal transducers outside the animal kingdom. Genome Biol..

[30] Usher KC (1998). Crystal structures of CheY from Thermotoga maritima do not support conventional explanations for the structural basis of enhanced thermostability. Protein Sci. Publ Protein Soc..

[31] Volz K, Beman J, Matsumura P (1986). Crystallization and preliminary characterization of CheY, a chemotaxis control protein from *Escherichia coli*. J. Biol. Chem..

[32] Swanson RV, Sanna MG, Simon MI (1996). Thermostable chemotaxis proteins from the hyperthermophilic bacterium Thermotoga maritima. J. Bacteriol..

[33] Correa A (2014). Potent and specific inhibition of glycosidases by small artificial binding proteins (affitins). PLoS ONE.

[34] Schilling J, Schöppe J, Plückthun A (2014). From DARPins to LoopDARPins: Novel LoopDARPin design allows the selection of low picomolar binders in a single round of ribosome display. J. Mol. Biol..

[35] Lopez-Hernandez E, Serrano L (1996). Structure of the transition state for folding of the 129 aa protein CheY resembles that of a smaller protein, CI-2. Fold Des..

[36] Kayushin AL (1996). A convenient approach to the synthesis of trinucleotide phosphoramidites–synthons for the generation of oligonucleotide/peptide libraries. Nucleic Acids Res..

[37] Popova B, Schubert S, Bulla I, Buchwald D, Kramer W (2015). A Robust and versatile method of combinatorial chemical synthesis of gene libraries via hierarchical assembly of partially randomized modules. PLoS ONE.

[38] Zemlin M (2003). Expressed murine and human CDR-H3 intervals of equal length exhibit distinct repertoires that differ in their amino acid composition and predicted range of structures. J. Mol. Biol..

[39] Tonegawa S (1983). Somatic generation of antibody diversity. Nature.

[40] Wilson IA, Stanfield RL (1994). Antibody-antigen interactions: New structures and new conformational changes. Curr. Opin. Struct. Biol..

[41] Padlan EA (1994). Anatomy of the antibody molecule. Mol. Immunol..

[42] Gilbreth RN, Koide S (2012). Structural insights for engineering binding proteins based on non-antibody scaffolds. Curr. Opin. Struct. Biol..

[43] Burg M (2004). Selection of internalizing ligand-display phage using rolling circle amplification for phage recovery. DNA Cell Biol..

[44] Christ D, Famm K, Winter G (2006). Tapping diversity lost in transformations—In vitro amplification of ligation reactions. Nucleic Acids Res..

[45] Freudl R (2018). Signal peptides for recombinant protein secretion in bacterial expression systems. Microb. Cell Fact..

[46] Bilwes AM, Alex LA, Crane BR, Simon MI (1999). Structure of CheA, a signal-transducing histidine kinase. Cell.

[47] Park SY, Beel BD, Simon MI, Bilwes AM, Crane BR (2004). In different organisms, the mode of interaction between two signaling proteins is not necessarily conserved. Proc. Natl. Acad. Sci. U.S.A..

[48] Inouye S, Sato J, Sahara-Miura Y, Yoshida S, Hosoya T (2014). Luminescence enhancement of the catalytic 19 kDa protein (KAZ) of Oplophorus luciferase by three amino acid substitutions. Biochem. Biophys. Res. Commun..

[49] Guellouz A (2013). Selection of specific protein binders for pre-defined targets from an optimized library of artificial helicoidal repeat proteins (alphaRep). PLoS ONE.

[50] Chevrel A (2018). Alpha repeat proteins (αRep) as expression and crystallization helpers. J. Struct. Biol..

[51] Urvoas A, Valerio-Lepiniec M, Minard P (2012). Artificial proteins from combinatorial approaches. Trends Biotechnol..

[52] Sikosek T, Chan HS (2014). Biophysics of protein evolution and evolutionary protein biophysics. J. R. Soc. Interface.

[53] Nangola S, Minard P, Tayapiwatana C (2010). Appraisal of translocation pathways for displaying ankyrin repeat protein on phage particles. Protein Expr. Purif..

[54] Fisher AC, Kim W, DeLisa MP (2006). Genetic selection for protein solubility enabled by the folding quality control feature of the twin-arginine translocation pathway. Protein Sci. Publ. Protein Soc..

[55] Speck J, Arndt KM, Müller KM (2011). Efficient phage display of intracellularly folded proteins mediated by the TAT pathway. Protein Eng. Des. Sel..

[56] Crameri A, Raillard SA, Bermudez E, Stemmer WP (1998). DNA shuffling of a family of genes from diverse species accelerates directed evolution. Nature.

[57] Simeon R, Chen Z (2018). In vitro-engineered non-antibody protein therapeutics. Protein Cell.

[58] Saito H, Minamisawa T, Shiba K (2007). Motif programming: a microgene-based method for creating synthetic proteins containing multiple functional motifs. Nucleic Acids Res..

[59] Scholle MD, Collart FR, Kay BK (2004). In vivo biotinylated proteins as targets for phage-display selection experiments. Protein Expr. Purif..

[60] Soltes G (2003). A new helper phage and phagemid vector system improves viral display of antibody Fab fragments and avoids propagation of insert-less virions. J. Immunol. Methods.

