# Supplementary material for: Design of an artificial phage-display library based on a new scaffold improved for average stability of the randomized proteins
Source: Sci Rep. 2023 Jan 24;13:1339. doi: 10.1038/s41598-023-27710-4 (PMC9873692; doi:10.1038/s41598-023-27710-4)
Supplement: Supplementary file 1 — Supplementary Information 1. [file 41598_2023_27710_MOESM1_ESM.pdf]

Design of an artificial phage-display library based on a new scaffold improved for average stability of the randomized proteins

Gomes, M. [1], Fleck, A. [1], Degaugue, A. [1], Gourmelon, F. [1], Léger, C.[1], Aumont-Nicaise, M. [1], Mesneau, A.[1], Jean-Jacques, H.[1], Hassaine, G. [2], Urvoas, A.[$ 1], Minard, P. [$ 1], Valerio-Lepiniec, M. [* $ 1]

[1] Université Paris-Saclay, CEA, CNRS, Institute for Integrative Biology of the Cell (I2BC), 91198, Gif-sur-Yvette, France.

[2]Arcoscreen, École Polytechnique Fédérale de Lausanne (EPFL), Lausanne Switzerland

[$] Equally contributed to this work;

*corresponding author: marielle.valerio@i2bc.paris-saclay.fr

Supplementary Information

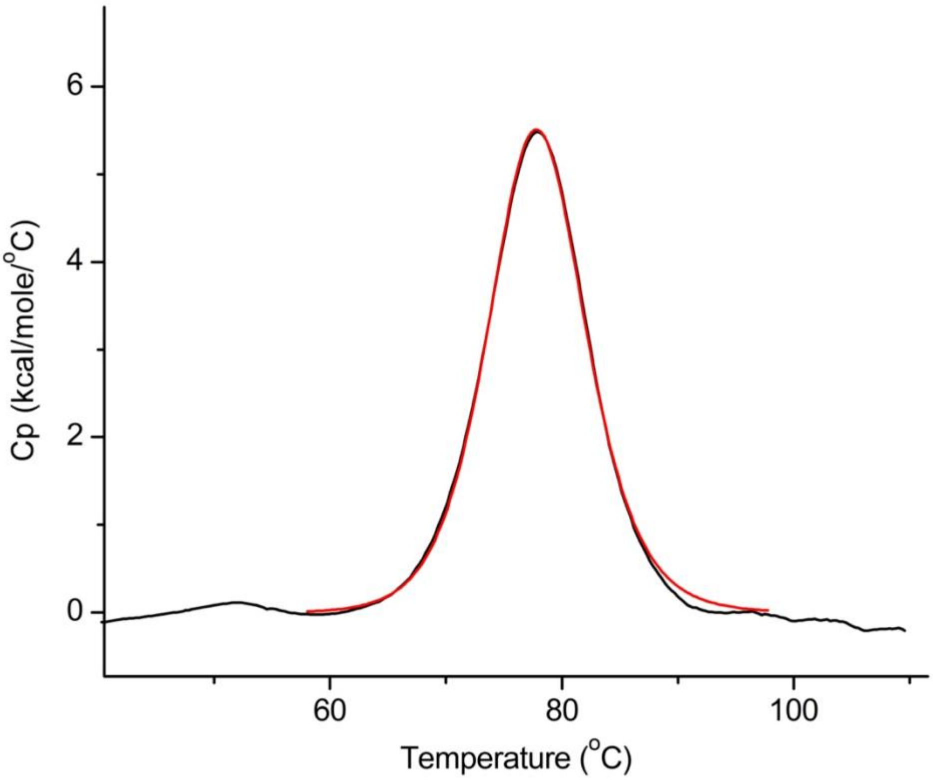

Figure S1: Heat denaturation of CheytinWT proteins assessed by DSC: Thermal stability was studied by differential scanning calorimetry (DSC) with a MicroCal VP-DSC instrument with CheytinWT at 1.75 mg.mL^{-1} in buffer Tris 20mM, $MgCl_2$ 5mM, NaCl 150mM pH 8.

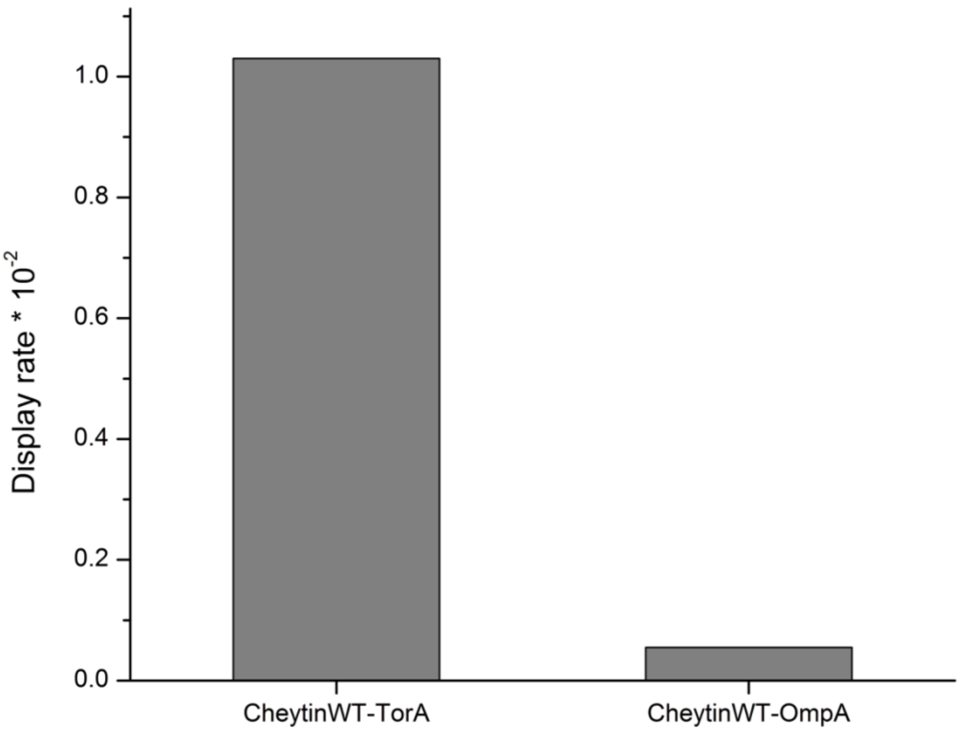

Figure S2: Cheytin's display rates onto M13 bacteriophage surface. Two bacterial secretion pathways such as SEC and TAT were tested to monitor the display efficiency of CheytinWT onto M13 bacteriophage surface. Phagemids containing the OmpA (SEC) or TorA (TAT) signal sequences were used to produce M13 bacteriophages displaying Cheytin scaffold. Phages containing the two populations of phagemids were produced using the protocol previously described[20]. The two populations of phages displaying the Cheytin scaffold with the OmpA and TorA were used to infect XL1-Blue MRF' bacteria to quantify the number of phages produced for each population (Input). The biotinylated version of P2 domain (His_6-P2-TEV-AviTag) was used to study the display rates of corrected folded Cheytins on the phages surface. The P2 domain was immobilised on the ELISA surface via the streptavidin-biotin interaction and the ELISA surface was blocked with TBS+3%BSA buffer to prevent unspecific binding. The exact same populations of phages were incubated in presence (specific retention) and in absence (non-specific retention) of the P2 domain pre-coated on an ELISA surface. After incubation, unbound phages were washed with TBST and TBS buffers. The remaining phages were specifically eluted with a TEV protease elution and used to infect XL1-Blue MRF' bacteria to quantify the number of phages retained with the P2 domain for each population (Specific output). The phages retained in absence of pre-coated P2 domain (Non-specific output) were also used to infect XL1-Blue MRF' bacteria to quantify the number of phages retained non-specifically during this experiment. The specific and non-specific display rates were calculated using the following formula: $Display\ Rate\ (\%) = \frac{Specific\ or\ Non\ Specific\ \ output\ Phages}{Input\ Phages} \times 100$. The non-specific display rate (3.9 10^{-6}) was negligeable as compared to the two specific rates. The TorA bacteriophages displaying the Cheytins via the TAT secretion pathway exhibited a 18-fold higher retention rate (1.03 10^{-2}) than the OmpA bacteriophages displaying the Cheytins via the SEC secretion pathway (0.055 10^{-2}). This results,

highlight the potential of the TAT system to export correctly folded proteins through the bacterial membrane.

Materials and Methods

Display rate measurement

100 µL/well of a solution of TBS (20 mM Tris-HCl pH 8.0, 150 mM NaCl) buffer containing 20 µg mL^{-1} of Streptavidin was used to coat the ELISA surface of a 96-well plate for 2 hours at 18°C and 300 rpm. After three washes with 300 µl/well of TBS containing 0,1% (V/V) of Tween-20 (TBST), the wells were blocked using 300µL/well of TBST supplemented with BSA 3% (W/V) overnight at 4°C and 300 rpm. 100 µL/well of a 40 µg mL^{-1} solution of the biotinylated version of P2 domain (His_6-P2-TEV-AviTag) was immobilised via the streptavidin-biotin interaction for 2 hours at 4°C and 300 rpm. After three washes with 300 µl/well of TBST, 100 µl/well of the OmpA or TorA phages were incubated in presence and in absence of the P2 domain for 2 hours at 4°C and 300 rpm. After 10 washes with TBST buffer and 10 washes with TBS buffer, phages were specifically eluted with 100 µl/well of a 10 µg mL^{-1} solution of TEV protease in 50 mM Tris pH 8.0, 0.5 mM EDTA et 1 mM DTT overnight at 4°C and 300 rpm. The initial solution of phages and the eluted phages were used to infect XL1-Blue MRF' bacteria and quantify the number of phages produced for each population (Input) and eluted in presence and in absence of the P2 domain (Output).

CheY

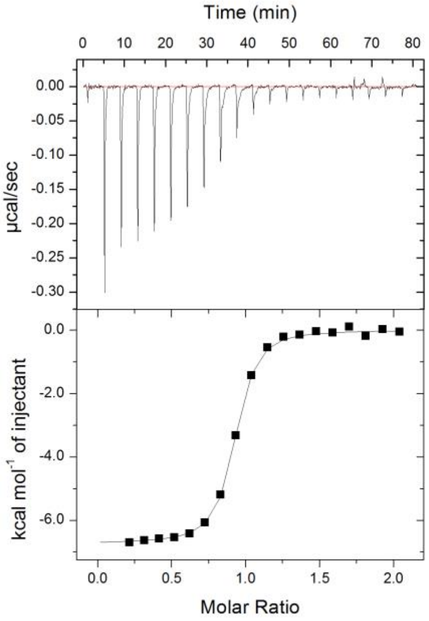

Cheytin-WT

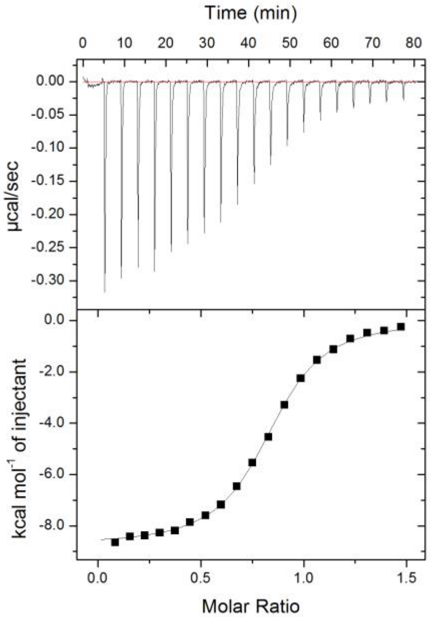

Cheytin 1

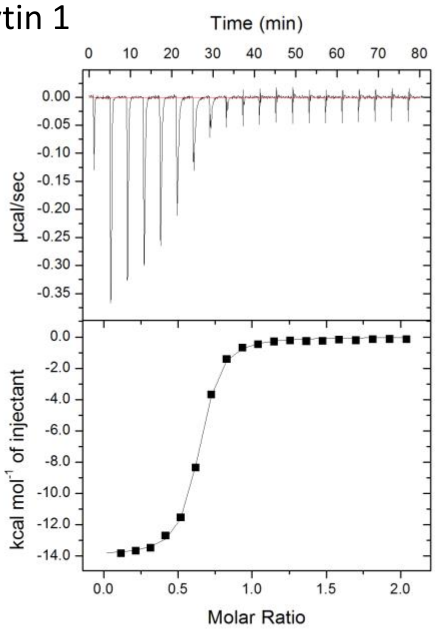

Cheytin 2

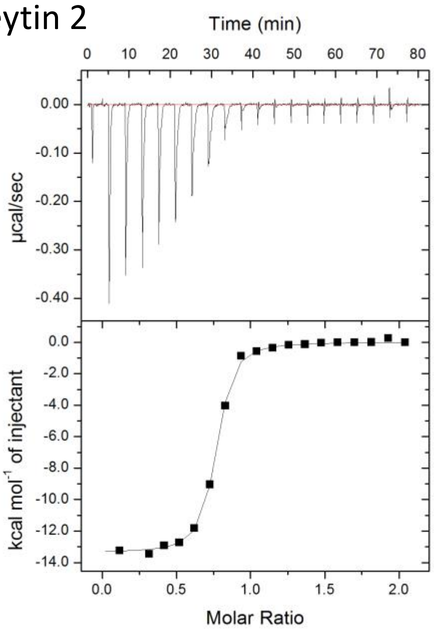

Cheytin 3

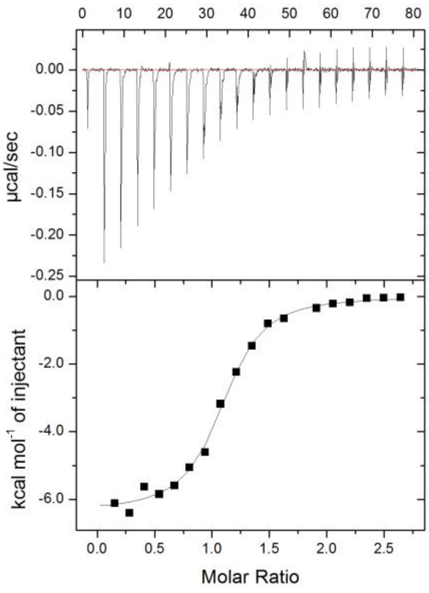

Cheytin 4

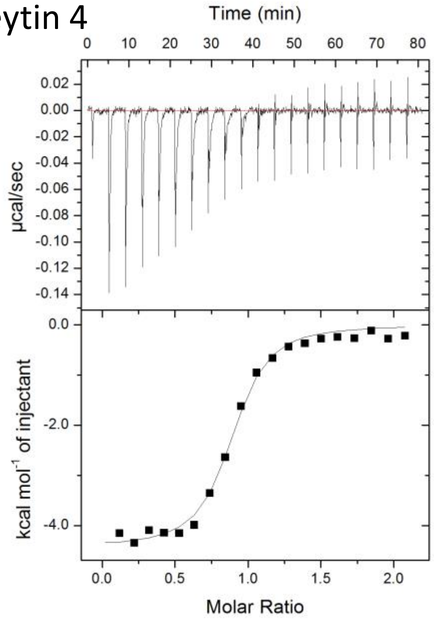

Clones	CheY	CheytinWT	Cheytin 1	Cheytin 2	Cheytin 3	Cheytin 4
K_D (μM)	0.15 ± 0,01	0.79 ± 0 .09	0.22 ± 0.05	0.11 ± 0.01	0.66 ± 0.09	0.49 ± 0.08
N	0.89 ± 0.003	0.82 ± 0.003	0.60 ± 0.002	0.73 ± 0.003	1.06 ± 0.001	0.86 ± 0.001

Figure S3: Interactions between CheY, CheytinWT and the Cheytins variants with the P2 protein monitored by ITC. For each ITC experiment, the raw data presented in the upper panel have been integrated in order to obtain the saturation curve presented in the lower panel. Parameters of each binding reaction, K_D and stoichiometry (N) are in the table presented below the ITC panel.

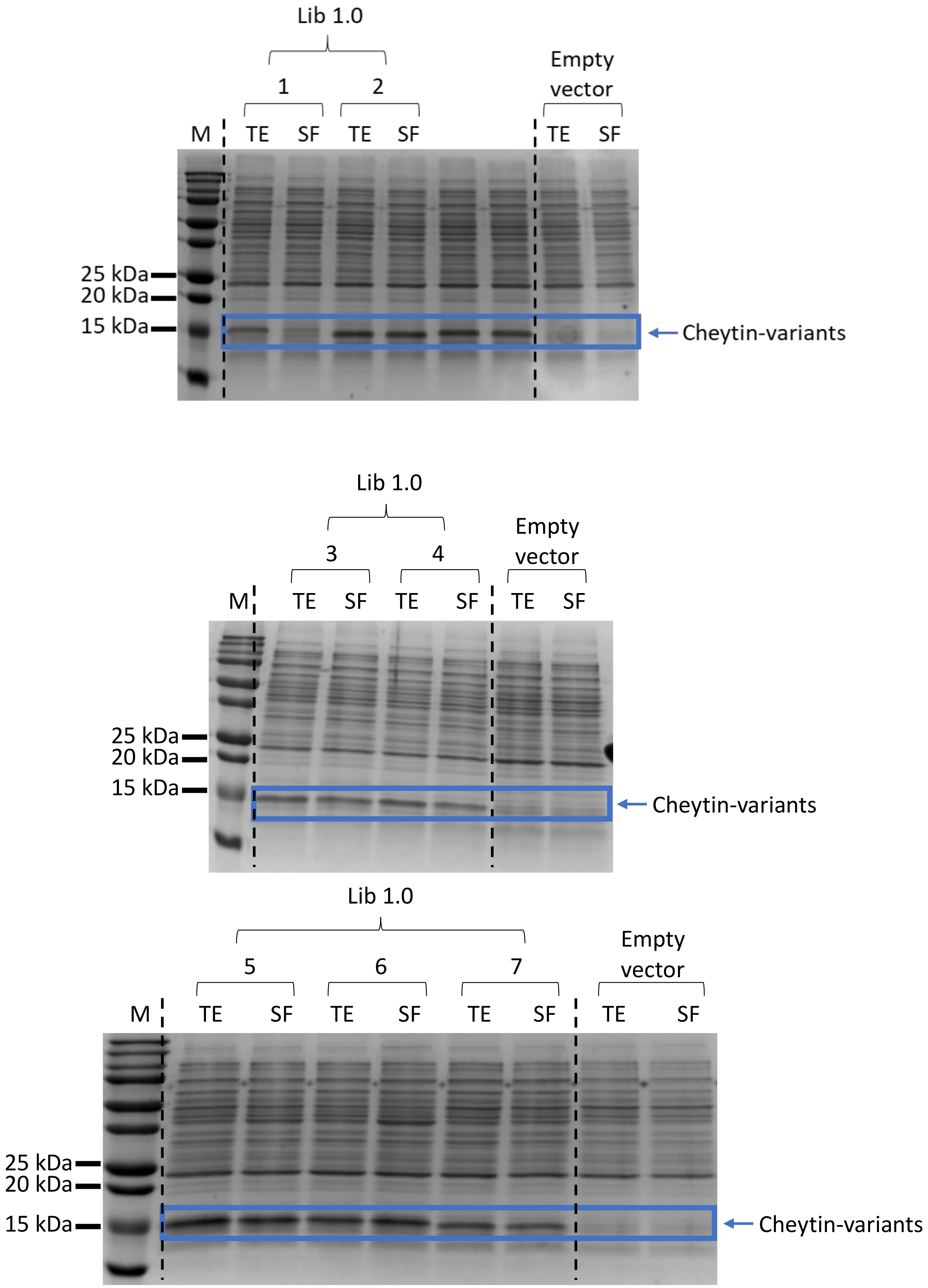

Lib 1.0
1
2
Empty vector
M
TE
SF
TE
SF
TE
SF
25 kDa
20 kDa
15 kDa
Cheytin-variants
Lib 1.0
3
4
Empty vector
M
TE
SF
TE
SF
TE
SF
25 kDa
20 kDa
15 kDa
Cheytin-variants
Lib 1.0
5
6
7
Empty vector
M
TE
SF
TE
SF
TE
SF
TE
SF
25 kDa
20 kDa
15 kDa
Cheytin-variants

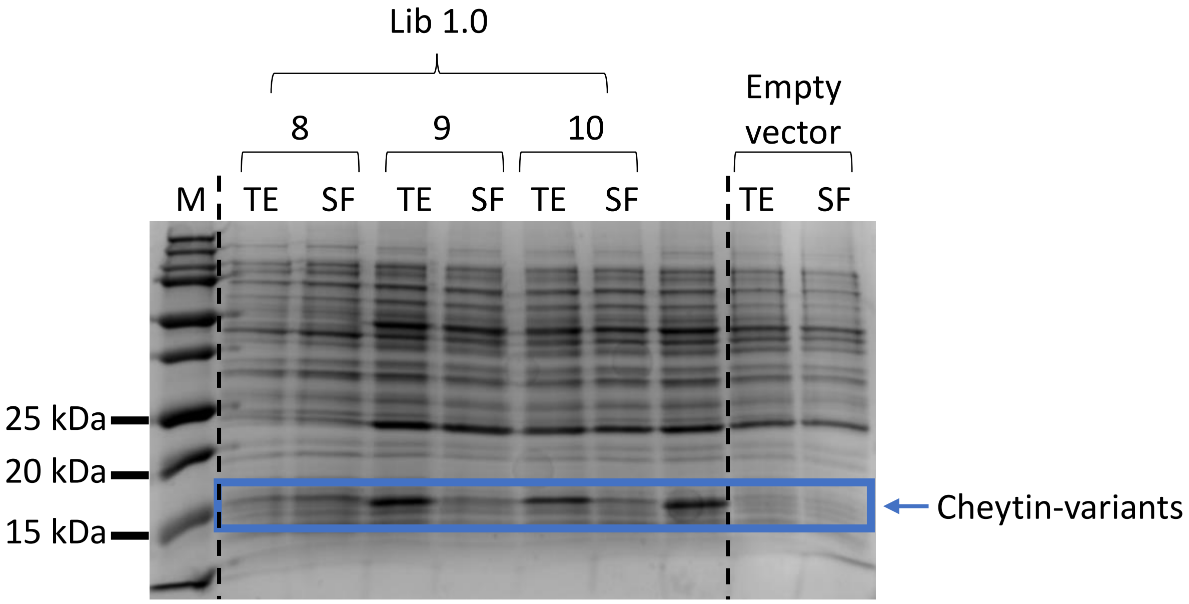
Lib 1.0
8
9
10
Empty vector
M
TE SF TE SF TE SF
TE SF
25 kDa
20 kDa
15 kDa
Cheytin-variants

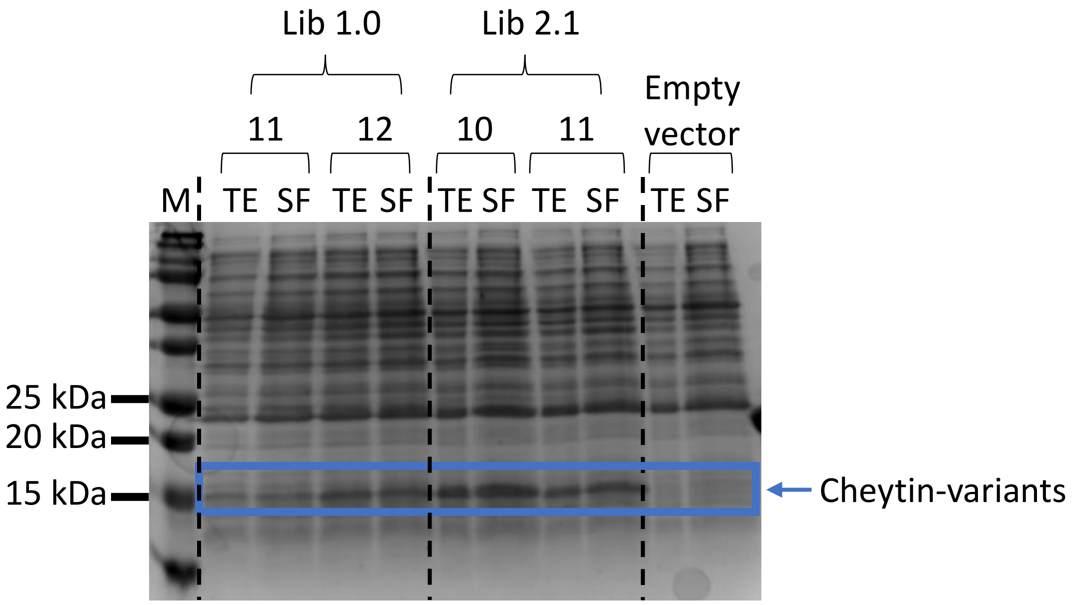
Lib 1.0
Lib 2.1
11
12
10
11
Empty vector
M
TE SF TE SF TE SF TE SF TE SF
25 kDa
20 kDa
15 kDa
Cheytin-variants

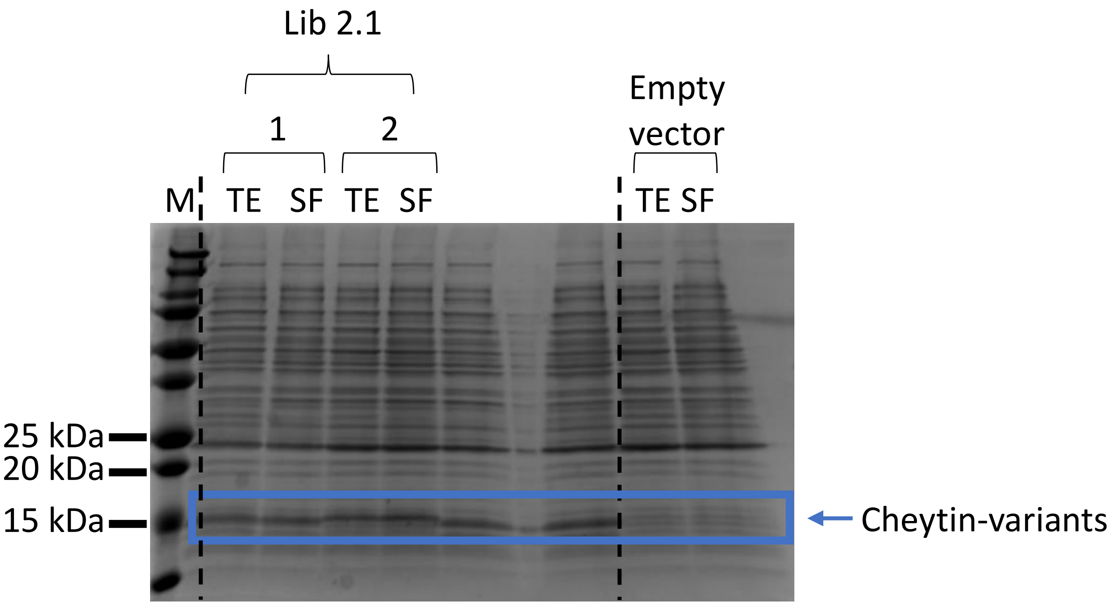
Lib 2.1
1
2
Empty vector
M
TE SF TE SF
TE SF
25 kDa
20 kDa
15 kDa
Cheytin-variants

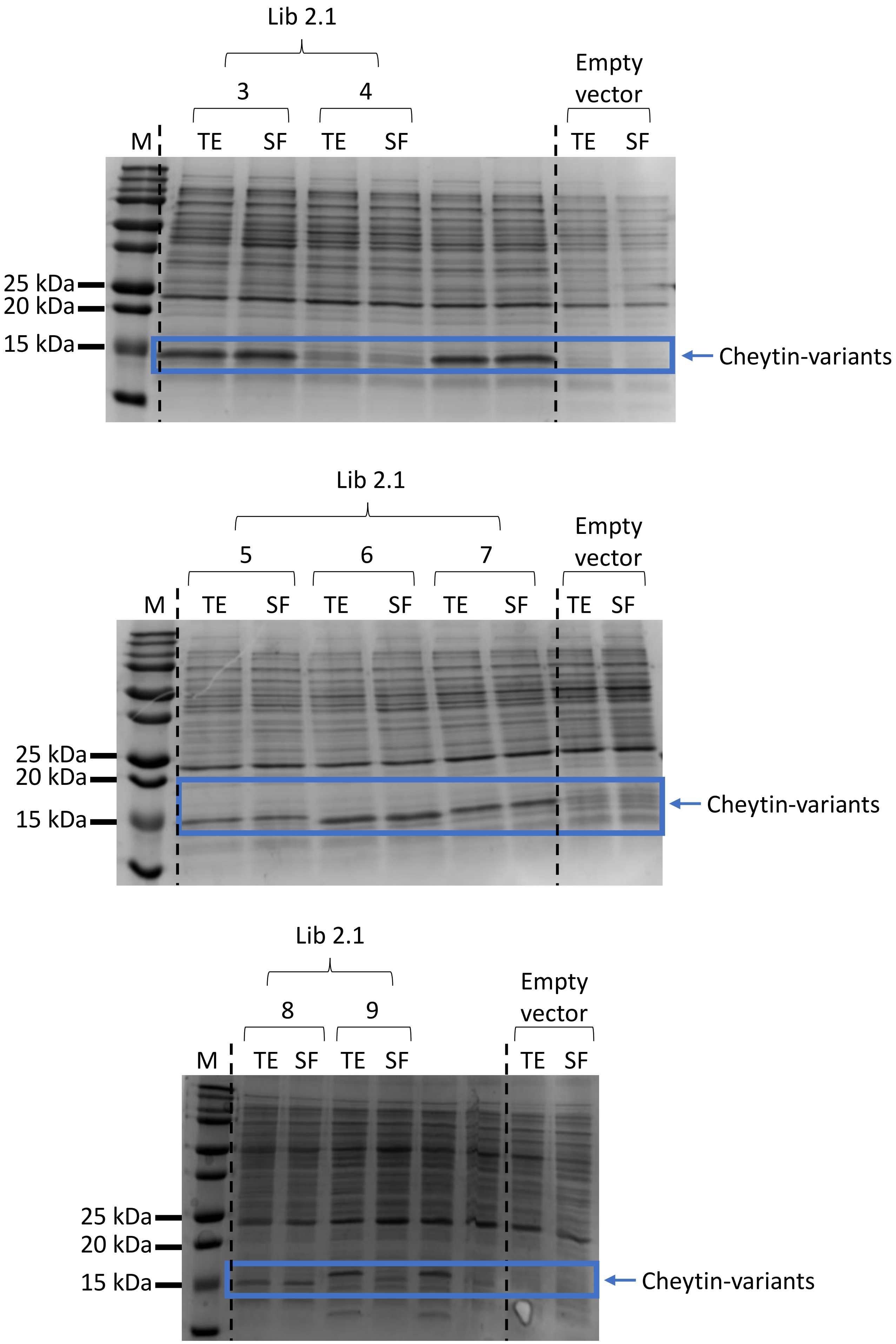
Lib 2.1
3
4
Empty vector
M
TE
SF
TE
SF
TE
SF
25 kDa
20 kDa
15 kDa
Cheytin-variants
Lib 2.1
5
6
7
Empty vector
M
TE
SF
TE
SF
TE
SF
TE
SF
25 kDa
20 kDa
15 kDa
Cheytin-variants
Lib 2.1
8
9
Empty vector
M
TE
SF
TE
SF
TE
SF
25 kDa
20 kDa
15 kDa
Cheytin-variants

Figure S4: Protein expression tests of the clones pooled for DSC experiment from Lib-Cheytins 1.0 and Lib-Cheytins 2.1

Analysis of protein expression of 12 clones from Lib-Cheytins 1.0 and 11 clones from Lib-Cheytins 2.1 by SDS-PAGE, stained with Coomassie blue. M: Molecular Weight markers; TE: total extract; SF: soluble fraction; Empty vector: control corresponding to expression level of a clone transformed with an empty vector (with no 2TMY-variant sequence). For all the clones, 2TMY protein variants were expressed and found in the soluble fraction compared to the empty vector.

Figure S4 regroups different full-length SDS-PAGE gels. For each gel, the image was acquired using the automated "Gel docTM EZ Imager Bio-Rad" imaging system; “Image lab 5.2.1” is the software used to scan each SDS-PAGE colored by Coomassie Blue. Key image-gathering settings and processing are the following: "Scanner publishing source; Image transform option: entire image; highlight saturated pixels; autoscale ». All images were recorded with the option “Export for publication” at 300 dpi resolution.

a)

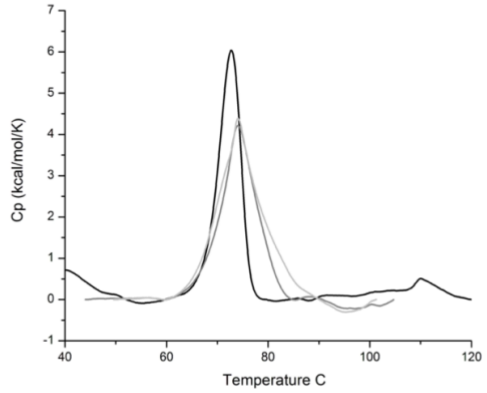

Clone Lib-Cheytins 1.0	Tm °C
2	74.27 ± 7.3 10^{-3}
5	74.33 ± 1.1 10^{-2}
7	72.43 ± 9.5 10^{-3}

b)

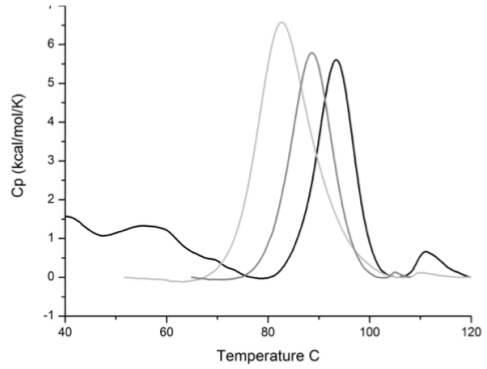

Clone Lib-Cheytins 2.1	Tm °C
1	83.34 ± 8.3 10^{-3}
3	88.33 ± 4.5 10^{-3}
9	93.29 ± 5.6 10^{-3}

Figure S5: Heat denaturation of individual Cheytin variants assessed by DSC: Thermal stability studied by DSC of **a)** 3 individual clones chosen among the 12 clones of the pool 1.0 (Clone 2, Clone 5, Clone 7) and **b)** 3 individual variants chosen among the 11 clones of the pool 2.1 (Clone 1, Clone 3, Clone 9) at 1.3 to 1.4 mg.mL^{-1} (depending on the experiment) in buffer Tris 20mM, MgCl2 5mM, NaCl 150mM pH 8.

a.

	1	2	3	4	5	6	7	8	9	10	11	12	Coating	Clones identification: selection round	plate position
A	0.062	0.250	0.066	0.065	0.065	0.065	0.067	0.067	0.065	0.066	0.066	0.072	S	1	PI -A
B	0.059	0.066	0.066	0.064	0.063	0.066	0.063	0.062	0.061	0.060	0.062	0.056	NS		
C	0.059	0.065	0.065	0.064	0.066	0.065	0.067	0.066	0.067	0.063	0.110	0.064	S	1	PI-B
D	0.064	0.063	0.061	0.058	0.059	0.062	0.063	0.064	0.063	0.058	0.064	0.063	NS		
E	0.377	0.253	0.460	0.253	0.310	0.240	0.343	0.234	0.196	0.081	0.206	0.212	S	2	PI-C
F	0.054	0.064	0.064	0.065	0.062	0.064	0.058	0.060	0.064	0.064	0.063	0.062	NS		
G	0.328	0.187	0.474	0.349	**0.445**	0.386	0.356	0.198	0.275	0.328	0.188	0.235	S	2	PI-D
H	0.050	0.060	0.063	0.061	**0.064**	0.062	0.063	0.063	0.063	0.060	0.064	0.062	NS		

	1	2	3	4	5	6	7	8	9	10	11	12	Coating	Clones identification: selection round	plate position
A	0.203	0.192	0.312	0.140	0.193	0.145	0.253	0.165	0.137	0.147	0.226	0.153	S	3	PI -E
B	0.066	0.063	0.064	0.067	0.063	0.062	0.065	0.063	0.063	0.064	0.063	0.064	NS		
C	0.299	0.292	0.272	0.343	**0.312**	0.233	0.304	0.173	0.238	0.247	0.259	0.212	S	3	PI-F
D	0.062	0.063	0.061	0.056	**0.056**	0.062	0.063	0.062	0.059	0.062	0.062	0.063	NS		
E	0.319	0.319	0.290	0.173	0.326	0.265	0.306	0.254	0.248	0.180	0.250	0.313	S	3	PI-G
F	0.059	0.061	0.061	0.062	0.061	0.062	0.063	0.059	0.059	0.062	0.061	0.061	NS		
G	0.306	0.287	0.249	0.266	0.210	0.288	0.255	0.260	0.279	0.327	0.284	0.211	S	3	PI-H
H	0.065	0.060	0.068	0.062	0.063	0.060	0.060	0.060	0.059	0.063	0.060	0.061	NS		

	1	2	3	4	5	6	7	8	9	10	11	12	Coating	Clones identification: selection round	plate position
A	0.248	0.154	0.283	0.266	0.163	0.123	0.188	0.203	0.289	0.184	**0.324**	0.309	S	3	PII -A
B	0.060	0.064	0.058	0.067	0.065	0.064	0.060	0.065	0.063	0.065	**0.065**	0.063	NS		
C	0.106	0.199	0.312	0.233	0.229	0.243	0.270	0.208	0.248	0.177	0.330	0.337	S	3	PII-B
D	0.058	0.063	0.060	0.063	0.059	0.059	0.062	0.061	0.060	0.062	0.064	0.061	NS		
E	0.262	0.330	0.265	0.164	0.211	0.169	0.228	0.203	0.232	0.165	0.238	0.234	S	3	PII-C
F	0.059	0.062	0.059	0.055	0.063	0.058	0.059	0.065	0.060	0.063	0.067	0.061	NS		
G	0.079	0.109	0.237	0.248	0.255	0.310	0.311	0.069	0.272	0.298	0.185	0.136	S	3	PII-D
H	0.056	0.060	0.063	0.062	0.062	0.059	0.061	0.062	0.061	0.060	0.064	0.060	NS		

	1	2	3	4	5	6	7	8	9	10	11	12	Coating	Clones identification: selection round	position
A	0.170	0.203	0.211	**0.243**	0.135	0.200	0.246	0.225	0.208	0.187	0.196	0.229	S	3	PII -E
B	0.061	0.063	0.065	**0.065**	0.067	0.064	0.065	0.064	0.063	0.064	0.063	0.062	NS		
C	0.162	0.113	0.199	0.184	0.200	0.220	0.252	0.179	0.193	0.217	0.080	0.247	S	3	PII-F
D	0.061	0.061	0.062	0.057	0.059	0.059	0.060	0.061	0.059	0.063	0.064	0.063	NS		
E	0.218	0.185	0.197	0.200	0.200	0.216	0.200	0.137	0.167	**0.230**	0.202	0.215	S	3	PII-G
F	0.053	0.059	0.057	0.061	0.057	0.056	0.058	0.061	0.056	**0.061**	0.061	0.061	NS		
G	0.196	0.164	0.204	0.136	0.182	0.226	0.200	0.160	0.177	0.221	0.178	0.231	S	3	PII-H
H	0.061	0.054	0.061	0.060	0.060	0.060	0.058	0.060	0.057	0.057	0.060	0.061	NS		

b.

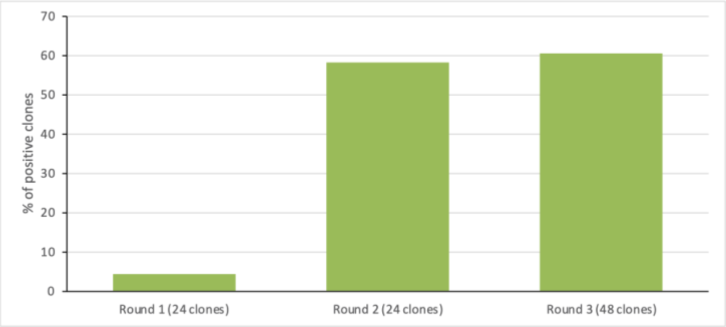

Figure S6. Phage-ELISA screening of binders from the Lib-Cheytin 2.1 library against the Kazα protein target.

Two plates of 96 clones (PI and PII) were screened after clonal phage production in deep-well plates. PI plate contained 24 clones from round 1, 24 clones from round 2 and 48 clones from round 3, and PII plate contained 96 clones from round 3. Phages from each clone were incubated on two wells of an ELISA plate to discriminate specific and non-specific signals: on a well with the biotinylated target immobilized on the coated streptavidin (row indicated S for specific) and a control well only coated with streptavidin and blocked with BSA (row indicated NS for non-specific). Positive clones were revealed with a horseradish peroxidase conjugated anti-M13 monoclonal antibody and detected at 450 nm using BM Blue POD as a substrate after the addition of HCl. **a)** Row absorbance (A) signals at 450 nm for the ELISA plates with each clone analyzed in two consecutive rows (S and NS). Clones that were further sequenced and characterized are indicated in red (PI: D5, F5 and PII: A11, E4, G10). **b)** Ratio of positive clones from plate I obtained after each round of selection. Clones were considered positive when the corrected signal (A-N) with superior to 3 times the noise signal ($S/N \geq 3$) with N = mean of non-specific signals for plate I ; $S/N = (A-N)/N$)

bKG10

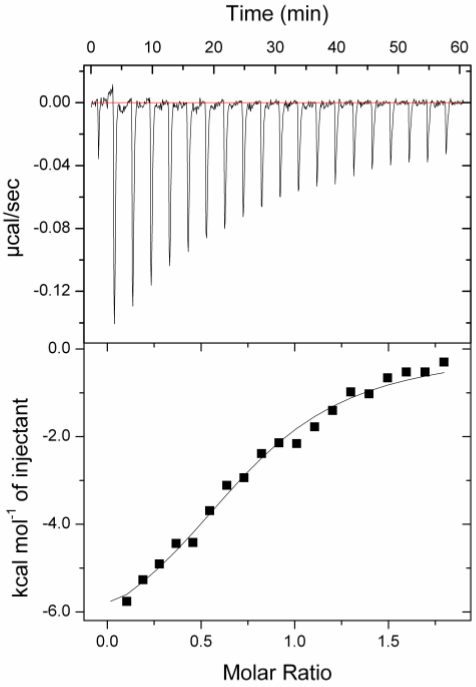

bKD5

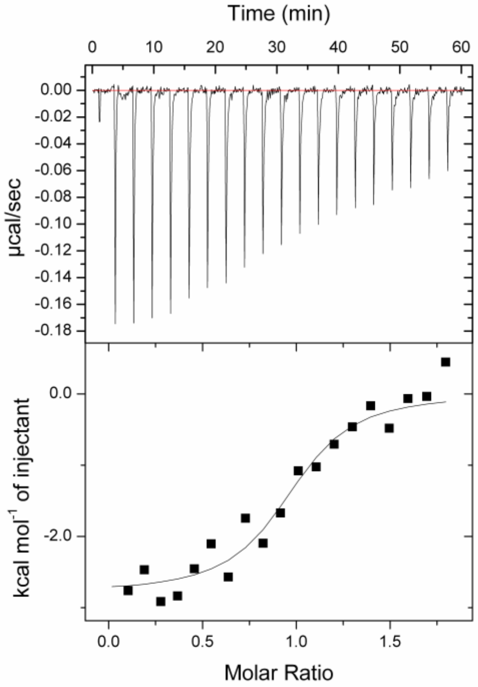

bKA11

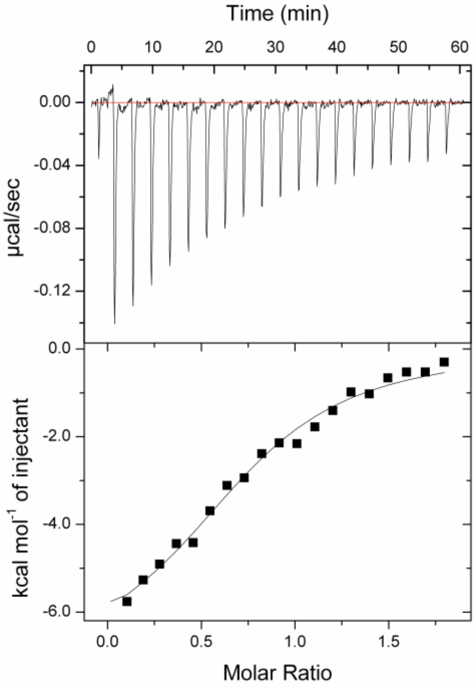

bKE4

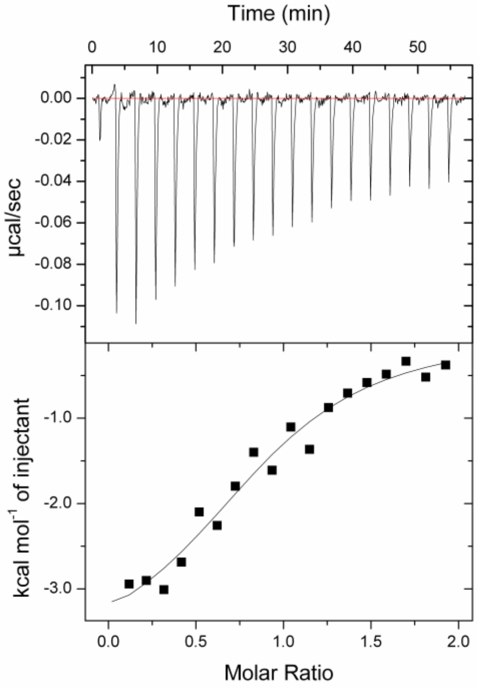

**Clones**	**bKG10**	**bKD5**	**bKA11**	**bKE4**
K_D (μM)	5.4 ± 0.7	0.77 ± 0 .3	4.8 ± 0.8	4.5 ± 1.3
N	0.83 ± 0.02	0.96 ± 0.04	0.75 ± 0.03	0.88 ± 0.06

Figure S7: Interaction between the Kazα binders and the Kazα, monitored by ITC. For each ITC experiment, the raw data presented in the upper panel have been integrated in order to obtain the saturation curve presented in the lower panel. Parameters of each binding reaction, Kd and stoichiometry (N) are presented in the table. Calorimetric titrations of Kazα (25μM) with binder bKG10 or bKD5 or bKA11 or bKE4, each at 220 μM.

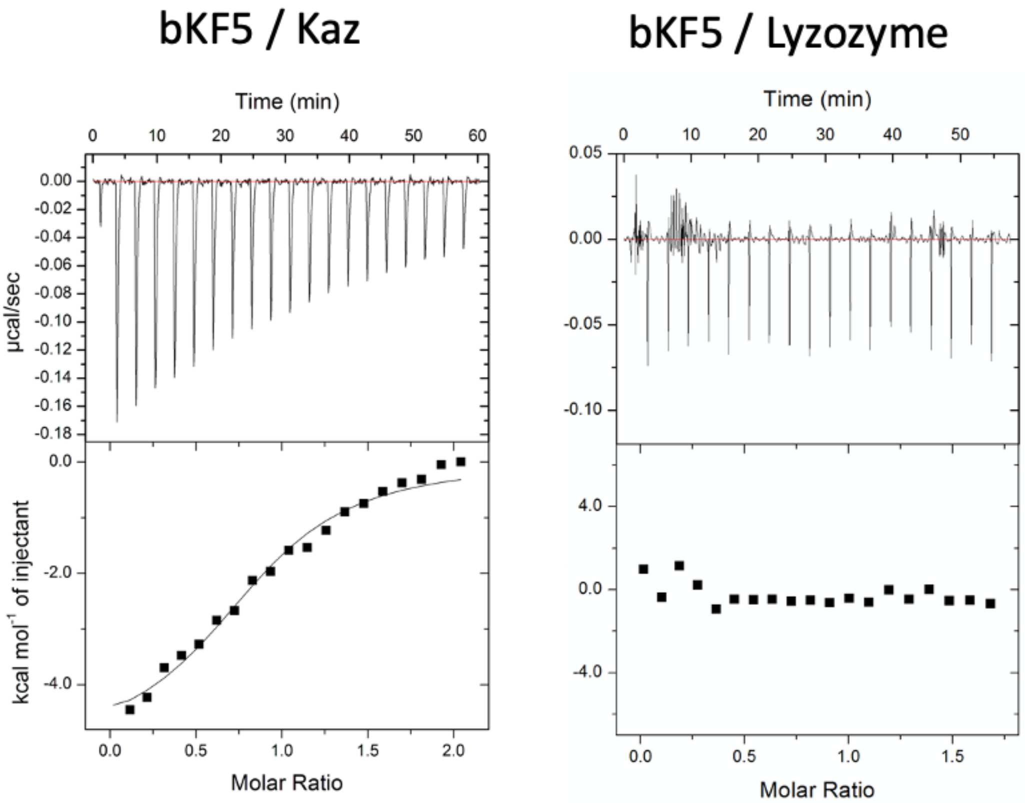

Figure S8: Specificity bF5K to Kazα monitored by ITC

Stoichiometry N = 0.85 ± 0.03 and K_D = 3.9 µM ± 0.7 obtained by ITC for the interactions between the Kazα and the bKF5. No interaction was observed between the Lysozyme, a non-relevant protein and the bKF5.

The binder bKF5 was in the syringe at 220 µM and the Lyzozyme or Kazα in the cell at 25 µM final concentration in the Cheytin buffer.
