# Supplementary material for: Design of an artificial phage-display library based on a new scaffold improved for average stability of the randomized proteins
Source: Sci Rep. 2023 Jan 24;13:1339. doi: 10.1038/s41598-023-27710-4 (PMC9873692; doi:10.1038/s41598-023-27710-4)
Supplement: Supplementary file 2 — Supplementary Information 2. [file 41598_2023_27710_MOESM2_ESM.pdf]

Design of an artificial phage-display library based on a new scaffold improved for average stability of the randomized proteins

Gomes, M. [1], Fleck, A. [1], Degaugue, A. [1], Gourmelon, F. [1], Léger, C.[1], Aumont-Nicaise, M. [1], Mesneau, A.[1], Jean-Jacques, H.[1], Hassaine, G. [2], Urvoas, A.[$ 1], Minard, P. [$ 1], Valerio-Lepiniec, M. [* $ 1]

[1] Université Paris-Saclay, CEA, CNRS, Institute for Integrative Biology of the Cell (I2BC), 91198, Gif-sur-Yvette, France.
[2]Arcoscreen, École Polytechnique Fédérale de Lausanne (EPFL), Lausanne Switzerland

[$] Equally contributed to this work;
*corresponding author: marielle.valerio@i2bc.paris-saclay.fr

Supplementary Tables

Table S1: GenBank accession numbers for the nucleotide sequences coding for the different proteins studied:

Experiment	Clone name mentioned in the article	GenBank accession numbers
12 Cheytins from Lib-Cheytins 1.0 pooled for DSC experiment	1<br>2<br>3<br>4<br>5<br>6<br>7<br>8<br>9<br>10<br>11<br>12	BankIt2604635 CHEYTIN1.0-3 OP158179<br>BankIt2604635 CHEYTIN1.0-4 OP158180<br>BankIt2604635 CHEYTIN1.0-5 OP158181<br>BankIt2604635 CHEYTIN1.0-7 OP158182<br>BankIt2604635 CHEYTIN1.0-8 OP158183<br>BankIt2604635 CHEYTIN1.0-9 OP158184<br>BankIt2604635 CHEYTIN1.0-12 OP158185<br>BankIt2604635 CHEYTIN1.0-13 OP158186<br>BankIt2604635 CHEYTIN1.0-18 OP158187<br>BankIt2604635 CHEYTIN1.0-19 OP158188<br>BankIt2604635 CHEYTIN1.0-22 OP158189<br>BankIt2604635 CHEYTIN1.0-24 OP158190
11 Cheytins from Lib-Cheytins 2.1 pooled for DSC experiment	1<br>2<br>3<br>4<br>5<br>6<br>7<br>8<br>9<br>10<br>11	BankIt2604266 CHEYTIN2.1-2 OP158168<br>BankIt2604266 CHEYTIN2.1-3 OP158169<br>BankIt2604266 CHEYTIN2.1-5 OP158170<br>BankIt2604266 CHEYTIN2.1-6 OP158171<br>BankIt2604266 CHEYTIN2.1-9 OP158172<br>BankIt2604266 CHEYTIN2.1-11 OP158173<br>BankIt2604266 CHEYTIN2.1-13 OP158174<br>BankIt2604266 CHEYTIN2.1-15 OP158175<br>BankIt2604266 CHEYTIN2.1-16 OP158176<br>BankIt2604266 CHEYTIN2.1-22 OP158177<br>BankIt2604266 CHEYTIN2.1-23 OP158178
P2-binders	Cheytin 1<br>Cheytin 2<br>Cheytin 3<br>Cheytin 4	BankIt2604652 CHEYTIN1 OP158191<br>BankIt2604652 CHEYTIN2 OP158192<br>BankIt2604652 CHEYTIN3 OP158193<br>BankIt2604652 CHEYTIN4 OP158194
Kaz-Binders	bKF5<br>bKG10<br>bKA11<br>bKD5<br>bKE4	BankIt2604652 BKF5 OP158195<br>BankIt2604652 BKG10 OP158196<br>BankIt2604652 BKA11 OP158197<br>BankIt2604652 BKD5 OP158198<br>BankIt2604652 BKAZE4 OP158199
Chimera used in luminescence study	CheytinWT-Kazα<br>bKF5-Kazα	BankIt2604652 CHEYTINWT-KAZALPHA OP158200<br>BankIt2604652 BKF5-KAZALPHA OP158201

Table S2: Relative amino acids frequencies in the randomized positions.

**Amino acids**	**Frequency (%)**
I	2.5
V	2.5
L	2.5
F	2.5
M	2.5
**A**	**6.5**
**G**	**18.5**
T	2.5
**S**	**8.5**
W	2.5
**Y**	**25**
P	2.5
H	2.5
Q	2.5
E	2.5
N	2.5
**D**	**6.5**
K	2.5
R	2.5
C	0
Stop	0
TOTAL	100

Table S3: Mass spectrometry analysis of proteins present in the pool from Lib-Cheytins 1.0 and Lib-Cheytins 2.1 used for the DSC experiment (see Figure 6):
Each protein pool was digested by trypsin. The resulting tryptic peptide pools were analyzed by nano LC-MS/MS with a DDA-PASEF method.
The identified sequences (in black font) and the protein sequence coverage are given for all the proteins. The randomized sequences of each protein are highlighted in yellow.

**Lib-Cheytins 1.0**	**Sequence identified by mass spectrometry**	**Protein sequence coverage (%)**
Cheytin 1.0-3	MRGSHHHHHHTDPRGKRVLIVDITRNMRMMLKDIITKAGYEVAGEATNGREAVEKYYELKPDIVTMDITYGYNGIRAIEDIMYIDPNAKIIVMSADMYQAMVIEAIKAGAKDFIVKYFYPSRVVEALNKVSKKLDYKDDDDKN	86
Cheytin 1.0-4	MRGSHHHHHHTDPRGKRVLIVDYNGNMRMMLKDIITKAGYEVAGEATNGREAVEKYYELKPDIVTMDITSKVSNGIRAIEDIMYIDPNAKIIVMSAYQDQAMVIEAIKAGAKDFIVKEFYPSRVVEALNKVSKKLDYKDDDDKN	89
Cheytin 1.0-5	MRGSHHHHHHTDPRGKRVLIVDYTGNMRMMLKDIITKAGYEVAGEATNGREAVEKYYELKPDIVTMDITGAYYNGIRAIEDIMYIDPNAKIIVMSAYYYQAMVIEAIKAGAKDFIVKYFDPSRVVEALNKVSKKLDYKDDDDKN	73
Cheytin 1.0-7	MRGSHHHHHHTDPRGKRVLIVDDMYNMRMMLKDIITKAGYEVAGEATNGREAVEKYYELKPDIVTMDITLDYDNGIRAIEDIMYIDPNAKIIVMSAYNGQAMVIGAIKAGAKDFIVKYFSPSRVVEALNKVSKKLDYKDDDDKN	73
Cheytin 1.0-8	MRGSHHHHHHTDPRGKRVLIVDYTPNMRMMLKDIITKAGYEVAGEATNGREAVEKYYELKPDIVTMDITHSTSNGIRAIEDIMYIDPNAKIIVMSADVTQAMVIEAIKAGAKDFIVKEFKPSRVVEALNKVSKKLDYKDDDDKN	93
Cheytin 1.0-9	MRGSHHHHHHTDPRGKRVLIVDHLKNMRMMLKDIITKAGYEVAGEATNGREAVEKYYELKPDIVTMDITMNYQNGIRAIEDIMYIDPNAKIIVMSADYRQAMVIEAIKAGAKDFIVKQFLPSRVVEALNKVSKKLDYKDDDDKN	84
Cheytin 1.0-12	MRGSHHHHHHTDPRGKRVLIVDWYSNMRMMLKDIITKAGYEVAGEATNGREAVEKYYELKPDIVTMDITYFKDNGIRAIEDIMYIDPNAKIIVMSADYIQAMVIEAIKAGAKDFIVKDFSPSRVVEALNKVSKKLDYKDDDDKN	93
Cheytin 1.0-13	MRGSHHHHHHTDPRGKRVLIVDGYGNMRMMLKDIITKAGYEVAGEATNGREAVEKYYELKPDIVTMDITGSNHNGIRAIEDIMYIDPNAKIIVMSAYYYQAMVIEAIKAGAKDFIVKTFHPSRVVEALNKVSKKLDYKDDDDKN	73
Cheytin 1.0-18	MRGSHHHHHHTDPRGKRVLIVDDYGNMRMMLKDIITKAGYEVAGEATNGREAVEKYYELKPDIVTMDITYYSFNGIRAIEDIMYIDPNAKIIVMSASYEQAMVIEAIKAGAKDFIVKGFGPSRVVEALNKVSKKLDYKDDDDKN	58
Cheytin 1.0-19	MRGSHHHHHHTDPRGKRVLIVDYYYNMRMMLKDIITKAGYEVAGEATNGREAVEKYYELKPDIVTMDITYYQGNGIRAIEDIMYIDPNAKIIVMSAYGYQAMVIEAIKAGAKDFIVKYFVPSRVVEALNKVSKKLDYKDDDDKN	ND
Cheytin 1.0-22	MRGSHHHHHHTDPRGKRVLIVDGDHNMRMMLKDIITKAGYEVAGEATNGREAVEKYYELKPDIVTMDITTPYHNGIRAIEDIMYIDPNAKIIVMSAGHYQAMVIEAIKAGAKDFIVKQFPPSRVVEALNKVSKKLDYKDDDDKN	73

Cheytin 1.0-24	MRGSHHHHHHTDPRGKRVLIVDMYYNMRMMLKDIITKAGYEVAGEATNGRE AVEKYYELKPDIVTMDITFHGYNGIRAIEDIMYIDPNAKIIVMSASSHQAM VIEAIKAGAKDFIVKYFNPSRVVEALNKVSKKLDYKDDDDKN	ND
**Lib-Cheytins 2.1**	**Sequence identified by mass spectrometry**	**Protein sequence coverage (%)**
Cheytin 2.1-2	MRGSHHHHHHTDPRGKRVLIVDADNNMRMMLKDIITKAGYEVAGEATNGRE AVEKYYELKPDIVTMDITYQYSNGIRAIEDIMYIDPNAKIIVMSAGGGQAM VIEAIKAGAKDFIVKDFRPSRVVEALNKVSKKLDYKDDDDKN	97
Cheytin 2.1-3	MRGSHHHHHHTDPRGKRVLIVDRYVNMRMMLKDIITKAGYEVAGEATNGRE AVEKYYELKPDIVTMDITGWGYNGIRAIEDIMYIDPNAKIIVMSASKYQAM VIEAIKAGAKDFIVKYFNPSRVVEALNKVSKKLDYKDDDDKN	89
Cheytin 2.1-5	MRGSHHHHHHTDPRGKRVLIVDSDMNMRMMLKDIITKAGYEVAGEATNGRE AVEKYYELKPDIVTMDITQYPYNGIRAIEDIMYIDPNAKIIVMSAYGDQAM VIEAIKAGAKDFIVKGFSPSRVVEALNKVSKKLDYKDDDDKN	97
Cheytin 2.1-6	MRGSHHHHHHTDPRGKRVLIVDGAGNMRMMLKDIITKAGYEVAGEATNGR EAVEKYYELKPDIVTMDITSGYPNGIRAIEDIMYIDPNAKIIVMSADGEQ AMVIEAIKAGAKDFIVKYFVPSRVVEALNKVSKKLDYKDDDDKN	92
Cheytin 2.1-9	MRGSHHHHHHTDPRGKRVLIVDRDGNMRMMLKDIITKAGYEVAGEATNGR EAVEKYYELKPDIVTMDITVKYANGIRAIEDIMYIDPNAKIIVMSAHTTQ AMVIEAIKAGAKDFIVKYFIPSRVVEALNKVSKKLDYKDDDDKN	89
Cheytin 2.1-11	MRGSHHHHHHTDPRGKRVLIVDADANMRMMLKDIITKAGYEVAGEATNGR EAVEKYYELKPDIVTMDITGNMSNGIRAIEDIMYIDPNAKIIVMSAKGYQ AMVIEAIKAGAKDFIVKPFDPSRVVEALNKVSKKLDYKDDDDKN	98
Cheytin 2.1-13	MRGSHHHHHHTDPRGKRVLIVDGMHNMRMMLKDIITKAGYEVAGEATNGR EAVEKYYELKPDIVTMDITYRAKNGIRAIEDIMYIDPNAKIIVMSASGGQ AMVIEAIKAGAKDFIVKSFGPSRVVEALNKVSKKLDYKDDDDKN	95
Cheytin 2.1-15	MRGSHHHHHHTDPRGKRVLIVDAYYNMRMMLKDIITKAGYEVAGEATNGR EAVEKYYELKPDIVTMDITFAGGNGIRAIEDIMYIDPNAKIIVMSAWGDQ AMVIEAIKAGAKDFIVKGFDPSRVVEALNKVSKKLDYKDDDDKN	92
Cheytin 2.1-16	MRGSHHHHHHTDPRGKRVLIVDGDRNMRMMLKDIITKAGYEVAGEATNGR EAVEKYYELKPDIVTMDITSLHDNGIRAIEDIMYIDPNAKIIVMSAIGGQ AMVIEAIKAGAKDFIVKDFYPSRVVEALNKVSKKLDYKDDDDKN	90
Cheytin 2.1-22	MRGSHHHHHHTDPRGKRVLIVDHYYNMRMMLKDIITKAGYEVAGEATNGR EAVEKYYELKPDIVTMDITYSYDNGIRAIEDIMYIDPNAKIIVMSAIGGQ AMVIEAIKAGAKDFIVKQFYPSRVVEALNKVSKKLDYKDDDDKN	92
Cheytin 2.1-23	MRGSHHHHHHTDPRGKRVLIVDGYYNMRMMLKDIITKAGYEVAGEATNGR EAVEKYYELKPDIVTMDITKYSYNGIRAIEDIMYIDPNAKIIVMSASGGQ AMVIEAIKAGAKDFIVKPFQPSRVVEALNKVSKKLDYKDDDDKN	96

Materials and Methods : Mass spectrometry

Protein pools from Lib-Cheytins 1.0 and Lib-Cheytins 2.1 used for the DSC experiment (in Tris 20 mM pH8, NaCl 200 mM, $MgCl_2$ 5 mM buffer) were diluted 7 fold in 50 mM Ammonium bicabonate buffer before overnight digestion at 37°C with sequencing-grade-modified trypsin (Promega, Madison, WI, USA) and using an enzyme-to-protein ratio between 1/5 and 1/20 (w/w). The resulting tryptic peptide pools were analyzed by nano LC-MS/MS using a nanoElute liquid chromatography system (Bruker, Billerica, MA, USA) coupled to a timsTOF Pro mass spectrometer (Bruker, Billerica, MA, USA). About 0.25 µg of protein digests in 1µL were loaded on an Aurora analytical column (ION OPTIK, 25 cm x 75 µm, C18, 1.6 µm) and eluted with a gradient of 0 to 35% of solvent B for 30 min (18 min from 0 to 15% B, 9 min from 15 to 23%B and 3 min from 23 to 35%B). Solvent A was 0.1% formic acid and 2% acetonitrile in water, and solvent B was 99.9% acetonitrile with 0.1% formic acid. MS and MS/MS spectra were recorded from m/z 100 to 1700 with a mobility scan range from 0.6 to 1.5 V.s/cm^2. MS/MS spectra were acquired with the PASEF (parallel accumulation-serial fragmentation (PASEF)) ion mobility-based and the data dependent acquisition mode using a number of PASEF MS/MS scans set as 10. MS and MSMS raw data were processed and converted into mgf files with Data Analysis software (Bruker, Billerica, MA, USA).
Protein identification was performed using the Mascot search engine against two homemade databases containing respectively the eleven or twelve protein sequences of the Lib-Cheytins 1.0 and Lib-Cheytins 2.1 pools. Database searches used trypsin cleavage specificity with two possible miscleavages and oxidation of methionines as variable modification. Peptide and fragment tolerances were set at 25 ppm and 0.05 Da, respectively.
